# Empowering Intelligent Surfaces and User Pairing for IoT Relaying Systems: Outage Probability and Ergodic Capacity Performance

**DOI:** 10.3390/s22176576

**Published:** 2022-08-31

**Authors:** Huu-Phuc Dang, Minh-Sang Van Nguyen, Dinh-Thuan Do, Minh-Hoa Nguyen, Minh-Triet Pham, Anh-Tuan Kim

**Affiliations:** 1 Electrical-Electronics Department, School of Engineering and Technology, Tra Vinh University, Tra Vinh 87000, Vietnam; 2 Faculty of Electronics Technology, Industrial University of Ho Chi Minh City (IUH), Ho Chi Minh City 70000, Vietnam; 3 Department of Computer Science and Information Engineering, College of Information and Electrical Engineering, Asia University, Taichung 41354, Taiwan

**Keywords:** reconfigurable intelligent surfaces, non-orthogonal multiple access, relay, outage probability, ergodic capacity

## Abstract

The evolution of Internet of Things (IoT) networks has been studied owing to the associated benefits in useful applications. Although the evolution is highly helpful, the increasing day-to-day demands of mobile users have led to immense requirements for further performance improvements such as efficient spectrum utilization, massive device connectivity, and high data rates. Fortunately, reconfigurable intelligent surfaces (RIS) and non-orthogonal multiple access (NOMA) techniques have recently been introduced as two possible current-generation emerging technologies with immense potential of addressing the above-mentioned issues. In this paper, we propose the integration of RIS to the existing techniques (i.e., NOMA and relaying) to further enhance the performance for mobile users. We focus on a performance analysis of two-user group by exploiting two main performance metrics including outage probability and ergodic capacity. We provide closed-form expressions for both performance metrics to highlight how NOMA-aided RIS systems provide more benefits compared with the benchmark based on traditional orthogonal multiple access (OMA). Monte-Carlo simulations are performed to validate the correctness of obtained expressions. The simulations show that power allocation factors assigned to two users play a major role in the formation of a performance gap among two users rather than the setting of RIS. In particular, the strong user achieves optimal outage behavior when it is allocated 35% transmit power.

## 1. Introduction

The evolution of sixth-generation (6G) wireless communication has added value to various applications. Consequently, this has led to a tremendous increase in the requirement of data connectivity and speed among users. Inefficient spectrum utilization and insufficient resources have demonstrated a major impact on the quality of services provided by previous generation networks. Thanks to recent technological advances, the enormous increase in efficiency has facilitated vast coverage for a huge number of users simultaneously. Though RIS were invented recently, their ability to perform signal transmissions similar to the smart radio environment (SRE) has rendered it a desirable mode of communication in many technologies [1]. There has been a huge number of articles reporting on its efficient performance in several technologies such as millimeter-Wave (mmWave), NOMA, OMA, cognitive radio (CR), etc. [2]. RIS is a meta-surface device that can control radio wave propagation by means of either reflecting or refracting radio signals based on the location of the user. In simple terms, the operation of RIS appears similar to a mirror reflection and refraction. Furthermore, RIS is a passive device meaning it does not add any external boost to the signal unlike the relaying technique.

The promising applications of RIS were discussed in recent studies [3,4,5,6,7,8,9,10,11,12]. In [3], the authors proposed RIS-based index modulation to improve spectral efficiency. The obtained results prove that this method also can provide high data rates with low error rates. In [4], the authors studied the free-space path loss model in RIS by validating the modeling results with mathematical results. RIS-aided multi-user multiple-input and multiple-output (MIMO) systems were proposed in [5] to estimate the cascaded channels of RIS. The authors proposed an alternating optimization algorithm to manage the non-convex problems and an estimation scheme which proved to be effective. The implementation of real-time RIS-based MIMO quadrature amplitude modulation was proposed in [6], which is a robust and cost-effective model with less energy-consuming hardware architecture. To enhance diverse transmission channel communication in a radio frequency (RF) sensing environment, the authors in [7] proposed a posture-recognizing RF-sensing system based on RIS. To improve the accuracy of the proposed model, optimization of various RIS factors were considered and the proposed model proved to be highly effective in recognition accuracy. In [8], the authors considered the RIS-aided multi-user multiple-input single-output (MISO) system to maximize the weighted sum-rate by beamforming at the access point and phase matrix design at RIS in both perfect and imperfect channel state information (CSI) scenarios. To obtain the matrix design, a low-complexity algorithm is designed utilizing fractional programming techniques. In [9], the authors considered the path loss of a channel consisting of reflecting array RIS using RIS size and link geometry. The secrecy performance of RIS in the presence of an eavesdropper is studied in [10], where the authors designed access point transmit beamforming and RIS reflect beamforming to maximize the secrecy rate of legitimate users. In [11], the authors examined the performance of RIS-aided point-to-point MIMO system and studied the characterization of the fundamental capacity of the RIS.

Upon inspection of the multiple access scheme applied to the RIS system, one can observe benefits of NOMA and OMA. In particular, the NOMA technique has become quite popular in recent times owing to its ability to share the same resource with more than one user [13]. In NOMA, two users are formed as a single cluster where the same resource slot is allocated to both users [14]. The signals of the users are transmitted by superposition coding and the received signals are separated by using the successive interference cancellation (SIC) technique [15]. Power allocation for the users plays a key role in differentiating the signals as the near user is allotted less power and the far user is allotted more power [16]. There are quite a few research works being published on the integration of NOMA with various technologies such as CR, massive-input massive-output [17], satellite communications [18], and under various scenarios such as perfect and imperfect SIC, CSI, hybrid NOMA. NOMA-based vehicle to everything (V2X) enabled backscatter communication is proposed in [19] to obtain optimal performance of the network compared to other traditional networks. Karush–Kuhn–Tucker (KKT) condition and sub-gradient methods are designed and the performance analyzed via Monte-Carlo simulations.

Several studies were performed suggesting the integration of RIS technology with NOMA as this combination can enhance the spectrum and energy efficiency, and increase the coverage area of signal transmission. In [20], the authors compared the performance of NOMA and OMA networks in the presence of the RIS downlink communication system. The goal was to minimize the transmit power by optimizing a few parameters in the network and a low-complexity solution was designed to obtain near-optimal performance. The numerical analysis shows that NOMA has a better performance capacity compared to its predecessor. In [21,22], the authors evaluated the throughput performance of the NOMA-assisted RIS system. In an effort to maximize the system throughput, joint optimization of the channel assignment, reflection coefficient, and power allocation was proposed by the authors through a low-complexity decoding order optimization algorithm. The simulation results showed that the correct placement of RIS can improve the throughput performance, along with the optimization technique. Outage and ergodic performance of RIS-assisted NOMA networks were studied in [23] in the presence of perfect and imperfect SIC and compared to OMA and relaying techniques. NOMA demonstrated superior performance over the other two techniques and also achieved enhanced energy efficiency among the network. Meanwhile, in [24], the authors proposed RIS-enhanced mmWave NOMA communications to enhance the sum rate of the system by jointly optimizing the active and passive beamforming and power allocation. To achieve optimization, the authors proposed alternating optimization and successive convex approximation-based iterative algorithm. In [25], the authors proposed an RIS-based NOMA system to implement more user allocation to the network compared to the spatial direction than the spatial division multiple access (SDMA) technique. Furthermore, the authors demonstrated the effectiveness of hardware impairments on the network and the simulations results showed that the proposed RIS-NOMA scheme had better user allocation.

## 2. The Related Works

### 2.1. Considerations of the Related Works

In [26], the authors proposed an RIS-assisted NOMA system for robust and secure communication via the introduction of artificial noise. It is assumed that the network has imperfect CSI of eavesdropper and transmit beamforming and phase shift optimization is performed using alternating optimization (AO) algorithm developed by the authors. Simulation analysis shows the advantage of a robust beamforming scheme AO algorithm in performing secure data communication through the network. In [27], RIS-assisted NOMA and OMA networks are considered with a cell-edge user device over a Nakagami-*m* fading channel. The performance of the system was analyzed and simulations showed the superior performance of RIS over decode and forward (DF) relay. A distributed RIS-enabled NOMA network was proposed in [28] to study the system’s secrecy performance in the presence of a passive eavesdropper. To maximize the minimum secrecy rate among the users, the authors considered jointly optimizing the transmit beamforming and phase shift at RIS. The authors proposed a ring-penalty-based successive convex approximation (SCA) algorithm and efficient AO algorithm. The numerical analysis showed the importance of the number of reflecting elements at the distributed RIS and the performance comparison between centralized and distributed RISs. In [29], the authors considered multiple unmanned aerial vehicle (UAV) mounted base station-assisted NOMA networks in the presence of an RIS and studied the performance of the system considering various parameters. To maximize the sum rate, the authors proposed joint optimization of the 3D placement, transmit power at UAV and reflection matrix at RIS, and decoding orders at NOMA. The authors proposed a block-decent coordinated-based algorithm and penalty-based SCA algorithm to obtain optimization of the parameters. The numerical analysis showed that optimization of the UAV placement can greatly increase the network performance and greatly enhance the channel quality served to the users. In [30], the authors studied the effect of implementing the hardware impairments as a real-time scenario in an RIS-assisted NOMA network. The numerical results and simulation analysis showed that the effect of hardware impairments can be considerably complemented when the number of meta-surfaces is sufficiently high. In [31], the authors presented the impact of coherent phase-shifting and random phase-shifting in NOMA-assisted RIS networks. To improve the reliability of the random phase-shifting method, the authors also proposed a low-complexity phase selection scheme.

We provide a comparison of our work with related works, shown in Table 1.

### 2.2. Motivations and Our Contributions

With regard to the aforementioned papers, there still remains a gap in the literature concerning investigations of the NOMA network in the presence of hybrid RIS and relay approach, as both differ in their operational characteristics but still performing similar functionality. The reason behind this is that the relay is still being implemented in emerging networks, while RIS is a new component requiring evaluation in terms of its synergistic functionality in conjunction with the relay. The relay might be installed in existing locations. However, the received signals are still weak in certain locations due to blocking or shadowing. Therefore, by enabling RIS at the walls of buildings, performance can be improved since RIS enhances the incident and reflecting signals targeting dedicated groups of users. It is worth pointing out that the limitations of this study regarding artificial intelligence and hardware impairments are not addressed since they are beyond the scope of this paper. Further, since one can limit interference to each user, we refer to each group of users containing only two users. More users in a group weaken the signal received at destinations. We will pursue Artificial Intelligence, multiple users and hardware impairments in future work. The contributions of this paper are as follows:

- We consider how RIS-NOMA and relay can work together to perform signal transmission to the two users in the framework over Rayleigh fading distribution and the network is considered as following perfect SIC and CSI.
- We first aim to clarify how the hybrid scheme exhibits some advantageous points compared with the related benchmark such as RIS-OMA. In particular, we simulate and determine main factors affecting RIS-NOMA to increase the effectiveness of processing.
- To conduct performance analysis, we introduce the closed-form expressions for outage probability (OP) and ergodic capacity (EC) for two representative users and some scenarios related to the presence of OMA, RIS and relay for comparison purposes.
- The numerical analysis can be performed via Monte-Carlo simulations to verify the validity of the obtained expressions. The simulations were performed to confirm the number of meta-surfaces at RIS, and the transmit SNR at the source are the main parameters affecting system performance.

The remainder of the paper is presented as follows. Section 3 describes the system model. Section 4 and Section 5 provide the OP and EC expressions of the users in the RIS-NOMA network. Section 6 provides the OPs and EC expressions of the users in the RIS-OMA network. Section 7 illustrates Figures for evaluation of the performance. Finally, concluding remarks are provided in Section 8.

## 3. System Model

**Figure 1:**
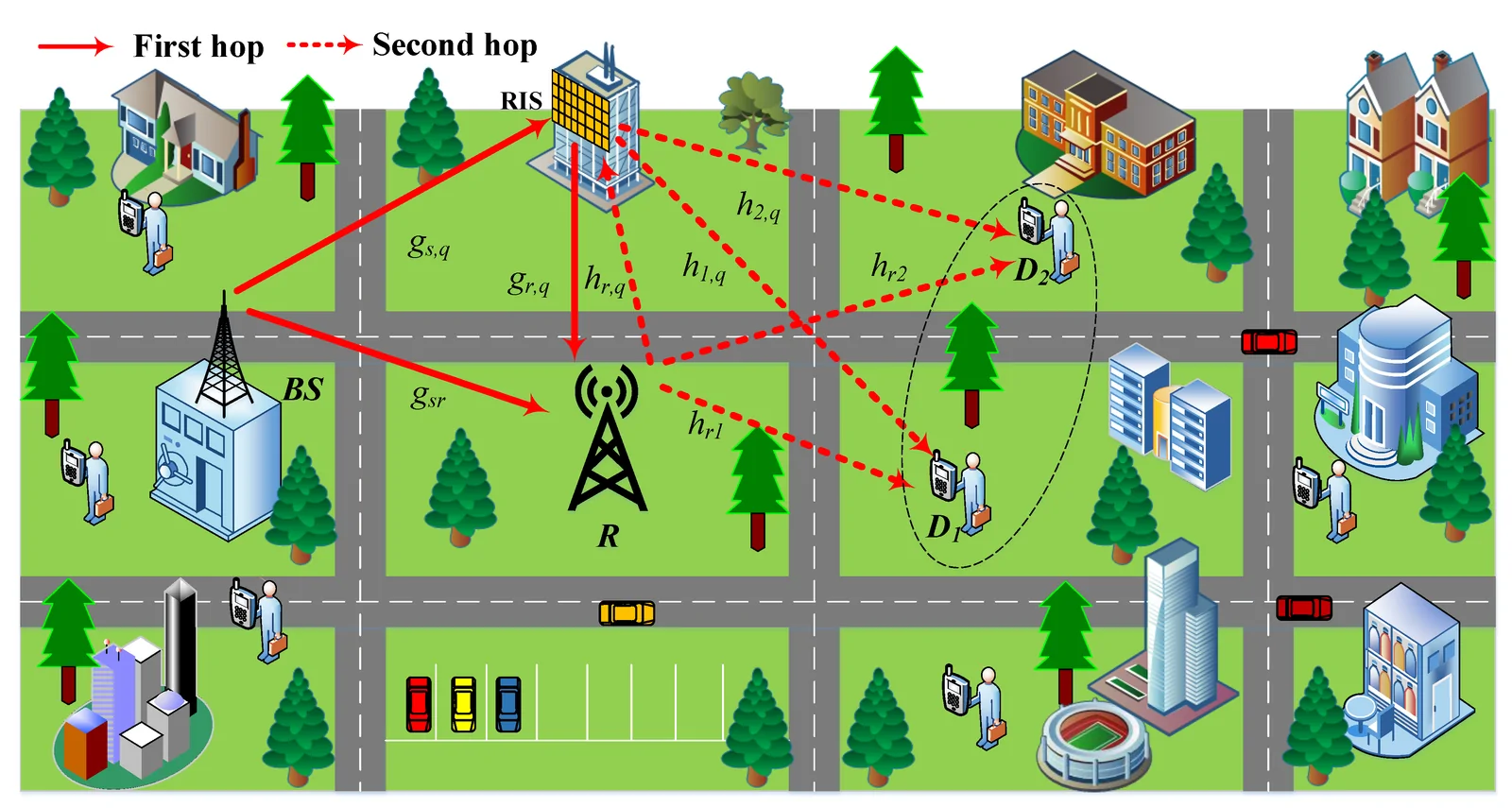
Hybrid relay RIS-aided IoT for downlink NOMA system.

As mentioned in Figure 1, we consider a RIS-NOMA network consisting of an access point or base station ( BS ), a relay (*R*), an RIS ( RI ), and two NOMA users $D_{1}$ and $D_{2}$ . It is noted that many IoT devices can be divided to many sets of users, and each set has two users. We could refer to more benefits from the joint design of a DF relay [42]. In the traditional IoT, a relay plays an important role in improving the performance for long-distance users and providing larger coverage areas. This paper considers RIS as a solution to foster these benefits for the existing system. Unlike the RI which has *Q* reflecting elements, BS , *R* and $D_{i}$ ( i=1,2 ) each have a single omni-directional antenna. It is assumed that BS and $D_{i}$ are located far apart from each other and there is no direct link between them owing to deep fading or obstructions [39,40]. Such transmission also requires two hops to deliver signals from BS to the destinations. During the first hop, BS transmits its signal to both *R* and RI , where the latter reflects the incident signal towards *R*. Therefore, the received signal at *R* can be given as [39,43]

(1) $$\begin{array}{c} y_{r}=\left(\frac{g_{sr}}{\sqrt{d_{sr}^{\varepsilon }}}+\sum_{q=1}^{Q}\frac{g_{s,q}g_{r,q}}{\sqrt{d_{si}^{\varepsilon }d_{ir}^{\varepsilon }}}\Omega_{q}e^{j\omega_{q}}\right)\left(\sqrt{P_{s}\chi_{1}}z_{1}+\sqrt{P_{s}\chi_{2}}z_{2}\right)+\delta_{r}. \end{array}$$

It is worth noting that the different demands of data service are required among two considered users, and hence we assume that $\chi_{2}>\chi_{1}$ with $\chi_{1}+\chi_{2}=1$ since BS adjusts the percentage of transmit antennas for each user [44]. $\Omega_{q}$ is the amplitude reflection coefficient with $\Omega_{q}\in \left(0,1\right]$ , $\omega_{q}$ is the adjustable phase applied by the *q*-th reflecting element with $\omega_{q}\in \left[0,2\pi \right]$ . In addition, $g_{sr}$ , $g_{s,q}$ , and $g_{r,q}$ are complex Gaussian random variables (RV) with zero mean and unit variance. The distances $d_{sr}$ , $d_{si}$ , $d_{ir}$ are denoted for the links BS−R , BS−RI and RI−R , respectively. With large *Q*, via the central limit theorem, we find that $\sum_{q=1}^{Q}g_{s,q}g_{r,q}∼CN\left(0,Q\right)$ and $g_{sr}∼CN\left(0,\lambda_{sr}\right)$ [45]. The other main parameters are shown in Table 2.

The received signal to interference plus noise ratio (SINR) at the relay to decode $z_{2}$ can be given as

(2) $$\gamma_{r}^{z_{2}}=\frac{\left(d_{sr}^{-\varepsilon }{\left|g_{sr}\right|}^{2}+\theta_{1}\Phi_{1}^{2}\right)\chi_{2}\varphi }{\left(d_{sr}^{-\varepsilon }{\left|g_{sr}\right|}^{2}+\theta_{1}\Phi_{1}^{2}\right)\chi_{1}\varphi +1}.$$

In order to simplify the analysis, Ideal Passive Beamforming (IPB) with Perfect Channel Estimation (PCE) is assumed at the RIS, and all elements have the same reflection amplitude. We have the phase $\omega_{q}=\arg\left(g_{sr}\right)-\arg\left(g_{s,q}g_{r,q}\right)$ and $\Omega_{q}=\Omega ,\forall_{q}$ [39], $\theta_{1}=\Omega^{2}d_{si}^{-\varepsilon }d_{ir}^{-\varepsilon }$ , $\varphi =\frac{P_{s}}{\sigma_{0}}$ , due to $\Phi_{1}=\left|\sum_{q=1}^{Q}g_{s,q}g_{r,q}e^{j\omega_{q}}\right|=\sum_{q=1}^{Q}\left|g_{s,q}\right|\left|g_{r,q}\right|$ in the case of perfect CSI [43,45]. It should be noted that perfect CSI is unattainable in practical scenarios. Therefore, the following analytical results can be treated as the upper bound of performance. We will deal with SIC and CSI imperfections in future work.

After conducting an SIC based on the principle of NOMA, the received SINR at the relay to decode $z_{1}$ can be given as

(3) $$\gamma_{r}^{z_{1}}=\left(d_{sr}^{-\varepsilon }{\left|g_{sr}\right|}^{2}+\theta_{1}\Phi_{1}^{2}\right)\chi_{1}\varphi .$$

*R* transmits the decoded signal to RI and $D_{1}$ , where RI reflects the incident signal towards D1 to be added constructively with the direct link from *R*. Therefore, after successful decoding of $z_{1}$ at *R*, the received signal at $D_{1}$ can be given as [39,43,45]

(4) $$\begin{array}{c} y_{D_{1}}=\left(\frac{h_{r1}}{\sqrt{d_{r1}^{\varepsilon }}}+\sum_{q=1}^{Q}\frac{h_{r,q}h_{1,q}}{\sqrt{d_{ri}^{\varepsilon }d_{i1}^{\varepsilon }}}\partial_{q}e^{j{℘}_{q}}\right)\left(\sqrt{P_{r}\chi_{1}}z_{1}+\sqrt{P_{r}\chi_{2}}z_{2}\right)+\delta_{1}, \end{array}$$

where $\partial_{q}$ is the amplitude reflection coefficient, ${℘}_{q}$ is the adjustable phase applied by the *q*-th reflecting element. $h_{r1}$ , $h_{r,q}$ , $h_{1,q}$ are complex Gaussian RV with zero mean and unit variance, $d_{r1}$ , $d_{ri}$ , $d_{i1}$ are the distances for the *R*- $D_{1}$ , *R*- RI and RI - $D_{1}$ links, respectively. We place our attention on Rayleigh distribution for these channels since we aim to characterize complete system performance metrics in closed-form expressions. Such results provide helpful guidelines to confirm the crucial role of RIS in its development in practical scenarios compared with the benchmark. Regarding Nakagami-m fading distributions for these channels, the readers are recommended to refer to the recent work in [12]. With large *Q*, via the central limit theorem, we find that $\sum_{q=1}^{Q}h_{r,q}h_{1,q}∼CN\left(0,Q\right)$ and $h_{r1}∼CN\left(0,\lambda_{r1}\right)$ [43,45].

The expected SINR at the user $D_{1}$ to decode $z_{2}$ can be formulated as

(5) $$\gamma_{D_{1}}^{z_{2}}=\frac{\left(d_{r_{1}}^{-\varepsilon }{\left|h_{r1}\right|}^{2}+\theta_{2}\Phi_{2}^{2}\right)\chi_{2}\varphi }{\left(d_{r_{1}}^{-\varepsilon }{\left|h_{r1}\right|}^{2}+\theta_{2}\Phi_{2}^{2}\right)\chi_{1}\varphi +1},$$

where in order to simplify the analysis, IPB with PCE is assumed at the RIS, and all elements have the same reflection amplitude. We have the phase ${℘}_{q}=\arg\left(h_{r1}\right)-\arg\left(h_{r,q}h_{1,q}\right)$ and $\partial_{q}=\partial ,\forall_{q}$ [39], $\varphi =\frac{P_{s}}{\sigma_{0}}=\frac{P_{r}}{\sigma_{0}}$ , $\theta_{2}=\partial^{2}d_{ri}^{-\varepsilon }d_{i1}^{-\varepsilon }$ , $\Phi_{2}=\left|\sum_{q=1}^{Q}h_{r,q}h_{1,q}e^{j{℘}_{q}}\right|=\sum_{q=1}^{Q}\left|h_{r,q}\right|\left|h_{1,q}\right|$ [43,45].

After SIC, the received SNR at user $D_{1}$ to decode $z_{1}$ can be given as

(6) $$\gamma_{D_{1}}^{z_{1}}=\left(d_{r_{1}}^{-\varepsilon }{\left|h_{r1}\right|}^{2}+\theta_{2}\Phi_{2}^{2}\right)\chi_{1}\varphi .$$

Similar to $D_{1}$ , the received signal at $D_{2}$ can be given as [39,43,45]

(7) $$\begin{array}{c} y_{D_{2}}=\left(\frac{h_{r2}}{\sqrt{d_{r2}^{\varepsilon }}}+\sum_{q=1}^{q}\frac{h_{r,q}h_{2,q}}{\sqrt{d_{ri}^{\varepsilon }d_{i2}^{\varepsilon }}}\varpi_{q}e^{j\kappa_{q}}\right)\left(\sqrt{P_{r}\chi_{1}}z_{1}+\sqrt{P_{r}\chi_{2}}z_{2}\right)+\delta_{2}, \end{array}$$

where $\varpi_{q}$ is the amplitude reflection coefficient, $\kappa_{q}$ is the adjustable phase applied by the *q*-th reflecting element. $h_{r2}$ , $h_{2,q}$ are complex Gaussian RV with zero mean and unit variance, $d_{r2}$ , $d_{i2}$ are the distances for the *R*- $D_{2}$ and RI - $D_{2}$ links, respectively. With large *Q*, via the central limit theorem, we find that $\sum_{q=1}^{Q}h_{r,q}h_{2,q}∼CN\left(0,Q\right)$ and $h_{r2}∼CN\left(0,\lambda_{r2}\right)$ [43,45].

The SINR at the user $D_{2}$ to decode $z_{2}$ can be formulated as

(8) $$\gamma_{D_{2}}^{z_{2}}=\frac{\left(d_{r2}^{-\varepsilon }{\left|h_{r2}\right|}^{2}+\theta_{3}\Phi_{3}^{2}\right)\chi_{2}\varphi }{\left(d_{r2}^{-\varepsilon }{\left|h_{r2}\right|}^{2}+\theta_{3}\Phi_{3}^{2}\right)\chi_{1}\varphi +1}.$$

Furthermore, IPB with PCE is assumed at the RIS, and all elements have the same reflection amplitude. We have the phase $\kappa_{q}=\arg\left(h_{r2}\right)-\arg\left(h_{r,q}h_{2,q}\right)$ and $\varpi_{q}=\varpi ,\forall_{q}$ [39], $\theta_{3}=\varpi^{2}d_{ri}^{-\varepsilon }d_{i2}^{-\varepsilon }$ , $\Phi_{3}=\left|\sum_{q=1}^{Q}h_{r,q}h_{2,q}e^{j\kappa_{q}}\right|=\sum_{q=1}^{Q}\left|h_{r,q}\right|\left|h_{2,q}\right|$ [43,45].

In the following sections, we shift our focus to system performance metrics, i.e., OP and EC, which provide meaningful guidelines to enable hybrid RIS and relay approach in practical applications.

## 4. OP Analysis

As mentioned in [13,14,15], we refer to evaluation of the main system performance metric, i.e., OP. In particular, an outage behavior of the considered system occurs when the achievable rate is less than the predetermined target rate.

### 4.1. OP of $D_{1}$

According to the NOMA protocol, the outage events of $D_{1}$ can be explained as below. The first is that *R* cannot detect $z_{1}$ . The second is that $D_{1}$ cannot detect its own message $z_{1}$ under the conditions that $D_{1}$ can detect $z_{2}$ successfully. Consequently, the OP of $D_{1}$ is expressed as [23,44]

(9) $$\begin{array}{c} OP_{D_{1}}=\mathrm{Pr}\left(\gamma_{r}^{z_{1}}<\vartheta_{1}\cup \gamma_{D_{1}}^{z_{2}}<\vartheta_{2}\cup \gamma_{D_{1}}^{z_{1}}<\vartheta_{1}\right)\\ =1-\mathrm{Pr}\left(\gamma_{r}^{z_{1}}\geq \vartheta_{1},\gamma_{D_{1}}^{z_{2}}\geq \vartheta_{2},\gamma_{D_{1}}^{z_{1}}\geq \vartheta_{1}\right)\\ =1-\underset{A_{1}}{\underset{︸}{\mathrm{Pr}\left(\gamma_{r}^{z_{1}}\geq \vartheta_{1}\right)}}\underset{A_{2}}{\underset{︸}{\mathrm{Pr}\left(\gamma_{D_{1}}^{z_{2}}\geq \vartheta_{2},\gamma_{D_{1}}^{z_{1}}\geq \vartheta_{1}\right)}}, \end{array}$$

where $\vartheta_{i}=2^{2R_{i}}-1,\left(i=1,2\right)$ .

**Proposition** **1.** *The closed-form expression for OP of the user $D_{1}$ is given by*

(10) $$\begin{array}{c} OP_{D_{1}}=1-\frac{d_{sr}^{-\varepsilon }\lambda_{sr}}{Q\theta_{1}-d_{sr}^{-\varepsilon }\lambda_{sr}}\exp\left(-\frac{\vartheta_{1}}{d_{sr}^{-\varepsilon }\varphi \beta_{1}\lambda_{sr}}\right)\left[\exp\left[\left(\frac{\theta_{1}}{d_{sr}^{-\varepsilon }\lambda_{sr}}-\frac{1}{Q}\right)\frac{\vartheta_{1}}{\varphi \beta_{1}\theta_{1}}\right]-1\right]\\ \times \frac{d_{r1}^{-\varepsilon }\lambda_{r1}}{\theta_{2}Q-d_{r1}^{-\varepsilon }\lambda_{r1}}\exp\left(-\frac{\nu }{d_{r1}^{-\varepsilon }\lambda_{r1}}\right)\left[\exp\left(\left(\frac{\theta_{2}}{d_{r1}^{-\varepsilon }\lambda_{r1}}-\frac{1}{Q}\right)\frac{\nu }{\theta_{2}}\right)-1\right], \end{array}$$

*where* $\nu =\max\left(\frac{\vartheta_{2}}{\left(\chi_{2}-\chi_{1}\vartheta_{2}\right)\varphi },\frac{\vartheta_{1}}{\chi_{1}\varphi }\right)$ .

**Proof.**  Please refer to Appendix A. □

**Remark** **1.** *It is worth noting that although many parameters affect the performance in (10), we can reference several main values to control the system performance. To achieve the evaluation of OP, these derivations can be performed in reliable and computationally efficient software packages, e.g., Mathematica, Matlab, where these values can be found accordingly. For example, if the locations of RIS, relay, and users can be determined, OP can be efficiently adjusted to satisfy requirements in the design of RIS-NOMA systems.*

### 4.2. OP of $D_{2}$

The outage events of $D_{2}$ can be explained two-fold. The first reason is that *R* cannot detect $z_{2}$ . The second reason is that $D_{2}$ cannot detect its own message $z_{2}$ . Based on these, the OP of $D_{2}$ is expressed as [23,44]

(11) $$\begin{array}{c} OP_{D_{2}}=\mathrm{Pr}\left(\gamma_{r}^{z_{2}}<\vartheta_{2}\cup \gamma_{D_{2}}^{z_{2}}<\vartheta_{2}\right)\\ =1-\mathrm{Pr}\left(\gamma_{r}^{z_{2}}\geq \vartheta_{2},\gamma_{D_{2}}^{z_{2}}\geq \vartheta_{2}\right)\\ =1-\underset{B_{1}}{\underset{︸}{\mathrm{Pr}\left(\gamma_{r}^{z_{2}}\geq \vartheta_{2}\right)}}\underset{B_{2}}{\underset{︸}{\mathrm{Pr}\left(\gamma_{D_{2}}^{z_{2}}\geq \vartheta_{2}\right)}}. \end{array}$$

**Proposition** **2.** *The closed-form expression for OP of the user $D_{2}$ is given by*

(12) $$\begin{array}{c} OP_{D_{2}}=1-\frac{d_{sr}^{-\varepsilon }\lambda_{sr}}{\theta_{1}Q-d_{sr}^{-\varepsilon }\lambda_{sr}}\exp\left(-\frac{\vartheta_{2}}{\left(\chi_{2}-\chi_{1}\vartheta_{2}\right)\varphi d_{sr}^{-\varepsilon }\lambda_{sr}}\right)\\ \times \left[\exp\left(\left(\frac{\theta_{1}}{d_{sr}^{-\varepsilon }\lambda_{sr}}-\frac{1}{Q}\right)\frac{\vartheta_{2}}{\left(\chi_{2}-\chi_{1}\vartheta_{2}\right)\varphi \theta_{1}}\right)-1\right]\\ \times \frac{d_{r2}^{-\varepsilon }\lambda_{r2}}{\theta_{3}Q-d_{r2}^{-\varepsilon }\lambda_{r2}}\exp\left(-\frac{\vartheta_{2}}{\left(\chi_{2}-\chi_{1}\vartheta_{2}\right)\varphi d_{r2}^{-\varepsilon }\lambda_{r2}}\right)\\ \times \left[\exp\left(\left(\frac{\theta_{3}}{d_{r2}^{-\varepsilon }\lambda_{r2}}-\frac{1}{Q}\right)\frac{\vartheta_{2}}{\left(\chi_{2}-\chi_{1}\vartheta_{2}\right)\varphi \theta_{3}}\right)-1\right]. \end{array}$$

**Proof.**  Please refer to Appendix B. □

**Remark** **2.** *These expressions of outage performance of the two different users can be adjusted to obtain their expectation when BS varies the main parameters such as power channel gains and power allocation factors $\chi_{1}$ , $\chi_{2}$ . Furthermore, the distances among these nodes affect the performance. We expect to evaluate how differences in performance of the two users can be achieved in the numerical results section.*

## 5. EC Analysis

If Quality of Service (QoS) requirements are set, the users’ data rates in the context of NOMA are opportunistically determined. In this section, ergodic capacity analysis is important to know since it defines the long-term average rate.

### 5.1. EC of $D_{1}$

On the condition that *R* and $D_{1}$ can detect $z_{1}$ , the achievable rate of $D_{1}$ can be written as ${ℜ}_{1}=\frac{1}{2}\log_{2}\left(1+\min\left(\gamma_{r}^{z_{1}},\gamma_{D_{1}}^{z_{1}}\right)\right)$ . The ergodic rate of $D_{1}$ can be obtained in the following [23,46,47]

(13) $$\begin{array}{cc} {\bar{ℜ}}_{1} & =E\left(\frac{1}{2}\log_{2}\left(1+\Xi_{1}\right)\right)\\ \; & =\frac{1}{2\ln2}\int_{0}^{\infty }\frac{1-F_{\Xi_{1}}\left(t\right)}{1+t}dt, \end{array}$$

where $\Xi_{1}=\min\left(\gamma_{r}^{z_{1}},\gamma_{D_{1}}^{z_{1}}\right)$ .

**Proposition** **3.** *The closed expression of ergodic rate for $D_{1}$ is given by*

(14) $${\bar{ℜ}}_{1}=-\frac{1}{2\ln2}\mu_{1}\mu_{2}\exp\left(\xi \right)\mathrm{Ei}\left(-\xi \right),$$

*where* $\mu_{1}=\frac{d_{sr}^{-\varepsilon }\lambda_{sr}}{d_{sr}^{-\varepsilon }\lambda_{sr}-Q\theta_{1}}$ , $\mu_{2}=\frac{d_{r1}^{-\varepsilon }\lambda_{r1}}{d_{r1}^{-\varepsilon }\lambda_{r1}-Q\theta_{2}}$ , $\xi =\frac{1}{d_{sr}^{-\varepsilon }\chi_{1}\varphi \lambda_{sr}}+\frac{1}{d_{r1}^{-\varepsilon }\chi_{1}\varphi \lambda_{r1}}$ .

**Proof.**  Please refer to Appendix C. □

### 5.2. EC of $D_{2}$

Since $z_{2}$ should be detected at *R* and $D_{2}$ as well as at $D_{1}$ for SIC, the achievable rate of $D_{2}$ is written as ${ℜ}_{2}=\frac{1}{2}\log_{2}\left(1+\min\left(\gamma_{r}^{z_{2}},\gamma_{D_{1}}^{z_{2}},\gamma_{D_{2}}^{z_{2}}\right)\right)$ . The corresponding ergodic rate is given by [23,46,47]

(15) $$\begin{array}{cc} {\bar{ℜ}}_{2} & =E\left(\frac{1}{2}\log_{2}\left(1+\Xi_{2}\right)\right)\\ \; & =\frac{1}{2\ln2}\int_{0}^{\infty }\frac{1-F_{\Xi_{2}}\left(t\right)}{1+t}dt, \end{array}$$

where $\Xi_{2}=\min\left(\gamma_{r}^{z_{2}},\gamma_{D_{1}}^{z_{2}},\gamma_{D_{2}}^{z_{2}}\right)$ .

**Proposition** **4.** *The exact expression of ergodic rate for $D_{2}$ is given by*

(16) $${\bar{ℜ}}_{2}=\frac{1}{2\ln2}\int_{0}^{1}\frac{\chi_{1}\chi_{2}\mu_{1}\mu_{2}\mu_{3}}{\left(\chi_{1}+\chi_{2}w\right)\chi_{1}}\exp\left(-\frac{\phi \chi_{2}w}{\left(\chi_{2}-\chi_{2}w\right)\chi_{1}}\right)dw,$$

*where* $\mu_{3}=\frac{d_{r2}^{-\varepsilon }\lambda_{r2}}{d_{r2}^{-\varepsilon }\lambda_{r2}-\theta_{3}Q}$ , $\phi =\frac{1}{\varphi d_{sr}^{-\varepsilon }\lambda_{sr}}+\frac{1}{\varphi d_{r1}^{-\varepsilon }\lambda_{r1}}+\frac{1}{\varphi d_{r2}^{-\varepsilon }\lambda_{r2}}$ .

**Proof.**  Please refer to Appendix D. □

### 5.3. The Asymptotic Expression for Ergodic Rate of $D_{2}$

From (16), by using the Gauss–Chebyshev integral [48,49], the asymptotic expression for ergodic rate of $D_{2}$ can provide the following integral approximation

(17) $$\begin{array}{c} {\bar{ℜ}}_{2}^{asym}=\frac{1}{2\ln2}\sum_{j=1}^{K}\frac{\pi \Lambda }{2K}\frac{\chi_{1}\chi_{2}\mu_{1}\mu_{2}\mu_{3}}{\left(\chi_{1}+\chi_{2}w_{j}\right)\chi_{1}}\exp\left(-\frac{\phi \chi_{2}w_{j}}{\left(\chi_{2}-\chi_{2}w_{j}\right)\chi_{1}}\right), \end{array}$$

where $w_{j}=\frac{1}{2}\left(1+\cos\left(\frac{2j-1}{G}\right)\right),\Lambda =\left|\sin\left(\frac{2j-1}{K}\right)\right|$ , *K* is the Gauss–Chebyshev integral approximated sum term [49].

**Remark** **3.** *It is worth noting that the EC performance presented in these derivations can be computed effectively if we use software packages, e.g., Mathematica, and Matlab, in which the values affecting EC can be found accordingly.*

## 6. Benchmark Scheme: RIS-OMA

**Figure 2:**
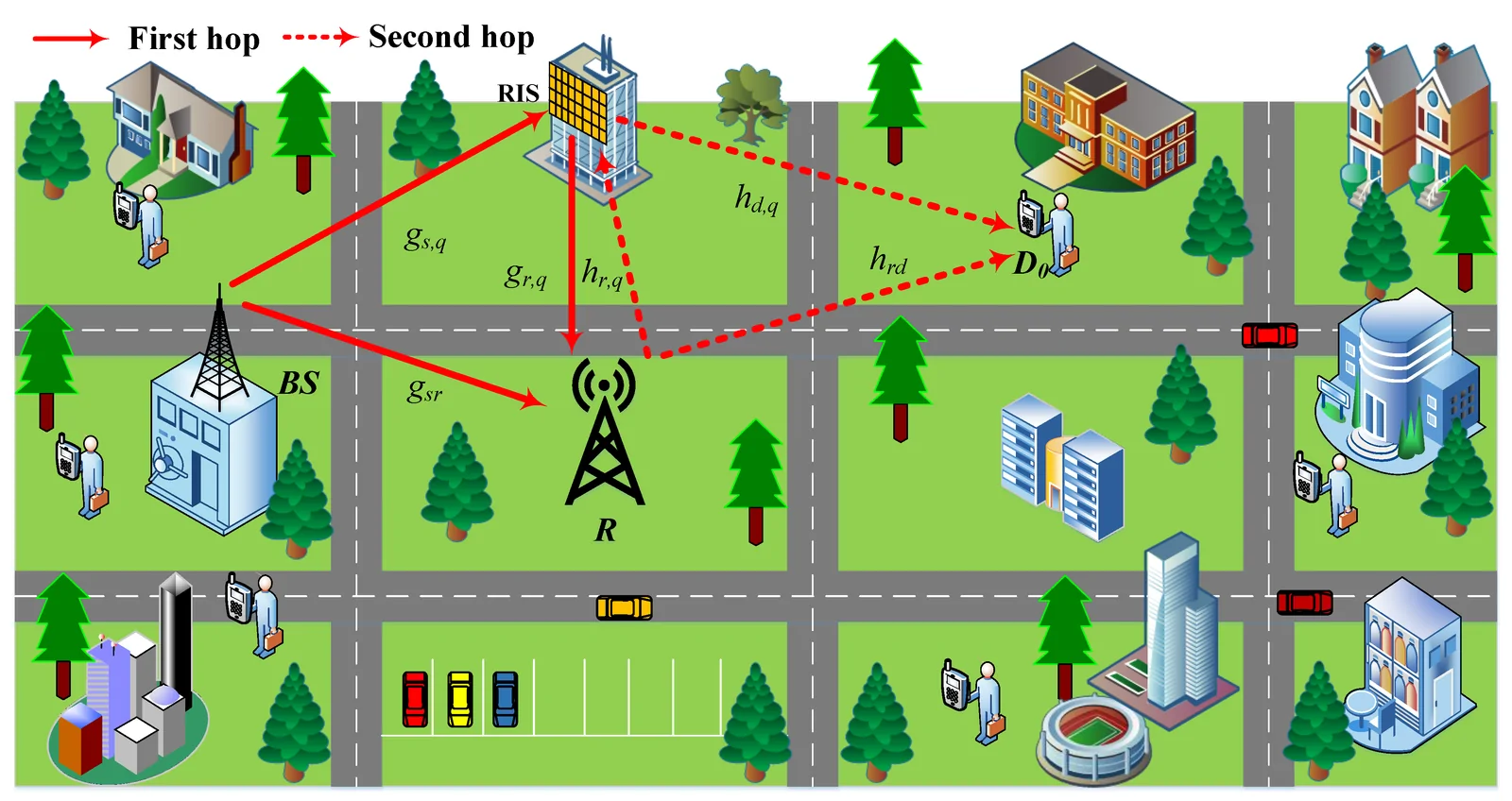
Hybrid relay RIS-OMA system.

In this subsection, the RIS-OMA scheme in Figure 2 is regarded as a necessary benchmark for comparison purposes, where an RI is deployed to assist in the transmission from the BS to a user $D_{o}$ [23]. Similar to (1), the received signal at *R* can be given as

(18) $$y_{r,o}=\left(\frac{g_{sr}}{\sqrt{d_{sr}^{\varepsilon }}}+\sum_{q=1}^{Q}\frac{g_{s,q}g_{r,q}}{\sqrt{d_{si}^{\varepsilon }d_{ir}^{\varepsilon }}}\Omega_{q}e^{j\omega_{q}}\right)\sqrt{P_{s}}z_{o}+\delta_{r}.$$

The received SNR at the relay to decode $z_{o}$ can be given as [23]

(19) $$\gamma_{r,o}=\left(d_{sr}^{-\varepsilon }{\left|g_{sr}\right|}^{2}+\theta_{1}\Phi_{1}^{2}\right)\varphi .$$

*R* transmits the decoded signal to RI and $D_{o}$ , where RI reflects the incident signal towards $D_{o}$ to be added constructively with the direct link from *R*. Therefore, after successful decoding of $z_{o}$ at *R*, the received signal at $D_{o}$ can be given as

(20) $$y_{D_{o}}=\left(\frac{h_{rd}}{\sqrt{d_{rd}^{\varepsilon }}}+\sum_{q=1}^{Q}\frac{h_{r,q}h_{d,q}}{\sqrt{d_{ri}^{\varepsilon }d_{id}^{\varepsilon }}}\partial_{q}e^{j{℘}_{q}}\right)\sqrt{P_{r}}z_{o}+\delta_{d},$$

where $h_{r,q}$ , $h_{d,q}$ are complex Gaussian RV with zero mean and unit variance, $d_{rd}$ , $d_{id}$ are the distances for the *R*- $D_{o}$ and RI - $D_{o}$ links, respectively. With large *Q*, via the central limit theorem, we find that $\sum_{q=1}^{Q}h_{r,q}h_{d,q}∼CN\left(0,Q\right)$ and $h_{rd}∼CN\left(0,\lambda_{rd}\right)$ [45].

The received SNR at user $D_{o}$ to decode $z_{o}$ can be given as [23]

(21) $$\gamma_{D_{o}}=\left(d_{rd}^{-\varepsilon }{\left|h_{rd}\right|}^{2}+\theta_{i,o}\Phi_{i,o}^{2}\right)\varphi ,$$

where in order to simplify the analysis, IPB with PCE is assumed at the RIS, and all elements have the same reflection amplitude. We have the phase ${℘}_{q}=\arg\left(h_{rd}\right)-\arg\left(h_{r,q}h_{d,q}\right)$ and $\partial_{q}=\partial ,\forall_{q}$ [39] $\theta_{i,o}=\partial^{2}d_{ri}^{-\varepsilon }d_{id}^{-\varepsilon }$ , $\Phi_{i,o}=\left|\sum_{q=1}^{Q}h_{r,q}h_{d,q}e^{j{℘}_{q}}\right|=\sum_{q=1}^{Q}\left|h_{r,q}\right|\left|h_{d,q}\right|$ [45].

### 6.1. Outage Performance Analysis

For RIS-OMA, an outage event is defined as the probability that the instantaneous SNR $\gamma_{r,o}$ and $\gamma_{D_{o}}$ fall below a threshold SNR $\vartheta_{o}$ . Hence, the OP of user $D_{o}$ can be expressed as [23]

(22) $$\begin{array}{cc} OP_{D_{o}} & =\mathrm{Pr}\left(\gamma_{r,o}<\vartheta_{o}\cup \gamma_{D_{o}}<\vartheta_{o}\right)\\ \; & =1-\underset{A_{1,o}}{\underset{︸}{\mathrm{Pr}\left(\gamma_{r,o}\geq \vartheta_{o}\right)}}\underset{A_{2,o}}{\underset{︸}{\mathrm{Pr}\left(\gamma_{D_{o}}\geq \vartheta_{o}\right)}}, \end{array}$$

where $\vartheta_{o}=2^{6R_{o}}-1$ .

**Proposition** **5.** *The closed-form expression for OP of the user* $D_{o}$ *is given by*

(23) $$\begin{array}{cc} OP_{D_{o}} & =1-\frac{d_{sr}^{-\varepsilon }\lambda_{sr}}{Q\theta_{1}-d_{sr}^{-\varepsilon }\lambda_{sr}}\frac{d_{rd}^{-\varepsilon }\lambda_{rd}}{Q\theta_{i,o}-d_{rd}^{-\varepsilon }\lambda_{rd}}\exp\left(-\frac{\vartheta_{o}}{d_{sr}^{-\varepsilon }\varphi \lambda_{sr}}-\frac{\vartheta_{o}}{d_{rd}^{-\varepsilon }\varphi \lambda_{rd}}\right)\\ \; & \times \left[\exp\left[\left(\frac{\theta_{1}}{d_{sr}^{-\varepsilon }\lambda_{sr}}-\frac{1}{Q}\right)\frac{\vartheta_{o}}{\varphi \theta_{1}}\right]-1\right]\left[\exp\left[\left(\frac{\theta_{i,o}}{d_{rd}^{-\varepsilon }\lambda_{rd}}-\frac{1}{Q}\right)\frac{\vartheta_{o}}{\varphi \theta_{i,o}}\right]-1\right]. \end{array}$$

**Proof.**  The details are given in Appendix E. □

### 6.2. Computation of EC

For RIS-OMA, based on (19) and (21), the ergodic rate of user $D_{o}$ can be expressed as [23,46]

(24) $$\begin{array}{cc} {\bar{ℜ}}_{O} & =E\left(\frac{1}{6}\log_{2}\left(1+\Xi_{O}\right)\right)\\ \; & =\frac{1}{6\ln2}\int_{0}^{\infty }\frac{1-F_{\Xi_{O}}\left(t\right)}{1+t}dt, \end{array}$$

where $\Xi_{O}=\min\left(\gamma_{r,o},\gamma_{D_{o}}\right)$ .

**Proposition** **6.** *The closed-form expression of ergodic rate for RIS-OMA is given by*

(25) $$\begin{array}{c} {\bar{ℜ}}_{O}=-\frac{1}{6\ln2}\frac{\mu_{1}d_{rd}^{-\varepsilon }\lambda_{rd}}{d_{rd}^{-\varepsilon }\lambda_{rd}-Q\theta_{i,o}}\exp\left(\frac{1}{d_{sr}^{-\varepsilon }\varphi \lambda_{sr}}+\frac{1}{d_{rd}^{-\varepsilon }\varphi \lambda_{rd}}\right)\mathrm{Ei}\left(-\left(\frac{1}{d_{sr}^{-\varepsilon }\varphi \lambda_{sr}}+\frac{1}{d_{rd}^{-\varepsilon }\varphi \lambda_{rd}}\right)\right). \end{array}$$

**Proof.**  The details are given in Appendix F. □

## 7. Numerical Results and Discussion

In this section, the numerical results are presented to confirm the rationality of the derived theoretical expressions for RIS-NOMA IoT networks. We show the impact of the reflecting elements on the performance of the RIS-assisted NOMA IoT network. In the simulation results, Rayleigh fading is assumed for all the channels [43]. The OP and EC are obtained via Monte Carlo simulations. The main parameters are presented in Table 3, excluding specific cases.

**Figure 3:**
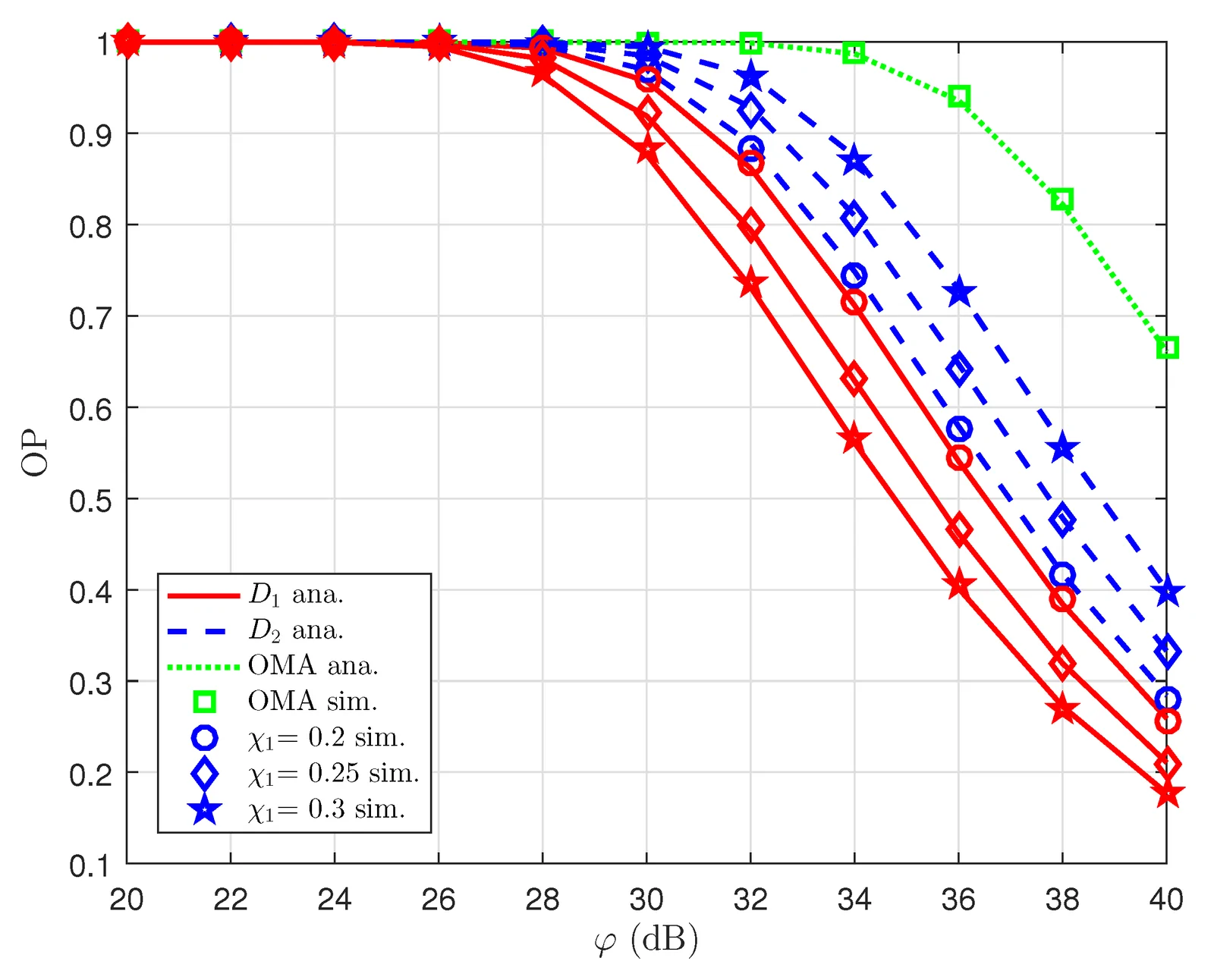
OP versus φ for different $\chi_{1}$ .

Figure 3 demonstrates the simulation of OP versus φ for different $\chi_{1}$ . As can be observed from the simulation, the OMA demonstrates the worst performance compared to both users in the NOMA network. As the φ increases, the outage performance of the IoT users increases relatively. The power level coefficient of the $D_{1}$ varies, and for the higher power level, the $D_{1}$ provides better outage performance and vice versa. The user $D_{1}$ with $\chi_{1}=0.3$ confirms its superior performance compared with the other cases.

**Figure 4:**
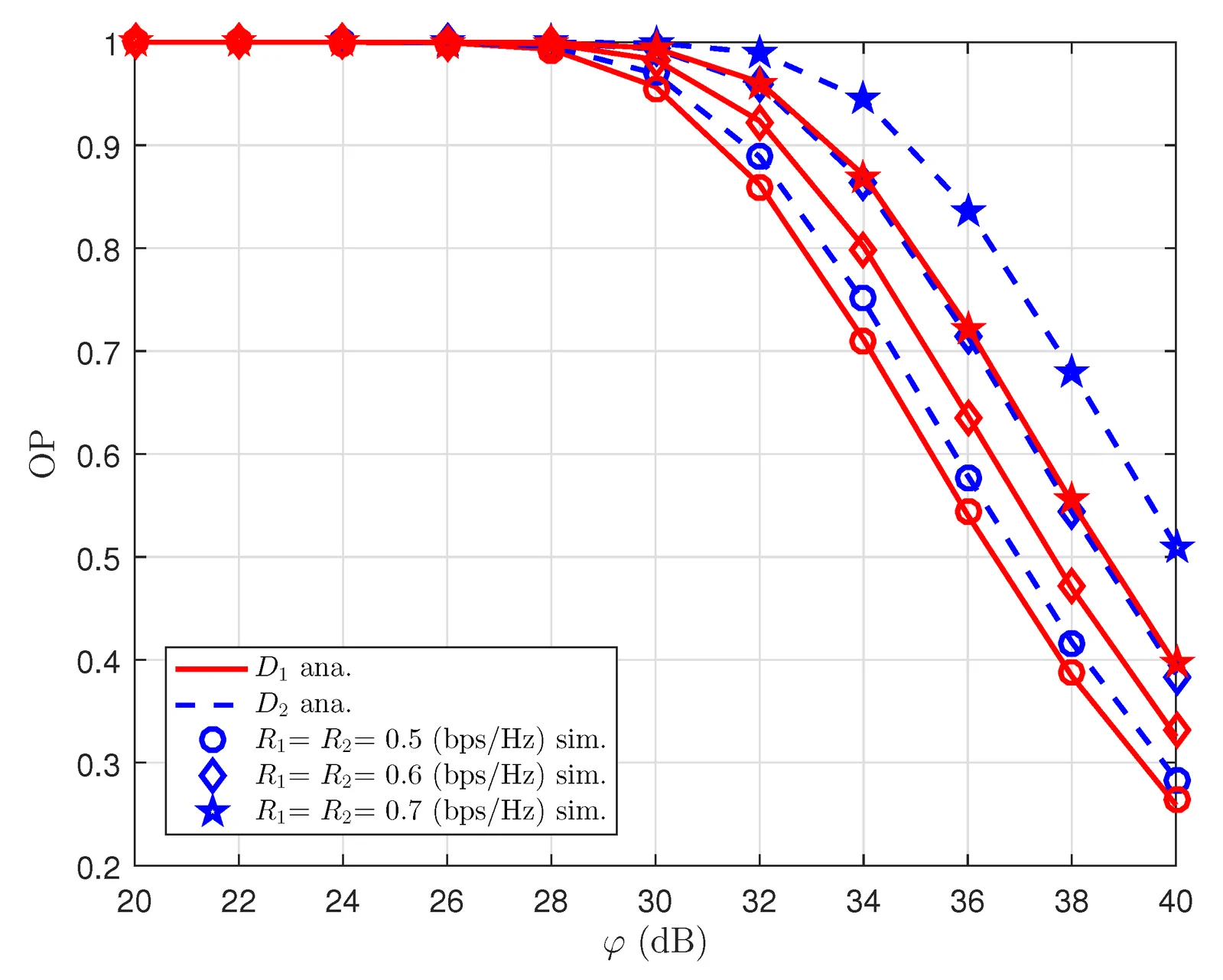
OP versus φ for different $R_{1}=R_{2}$ .

Figure 4 exhibits the simulation of OP versus φ for different $R_{1}=R_{2}$ . It is observed from the simulation that as the target rates of the users are increased, the performance is reduced. With the increase in transmit SNR, the performance of both users increases relatively and the gap between the curves of the two users is maintained for all cases of target rates. The lower requirement of target rate results in better outage behavior, i.e., $R_{1}=R_{2}=0.5$ is reported as the best case.

**Figure 5:**
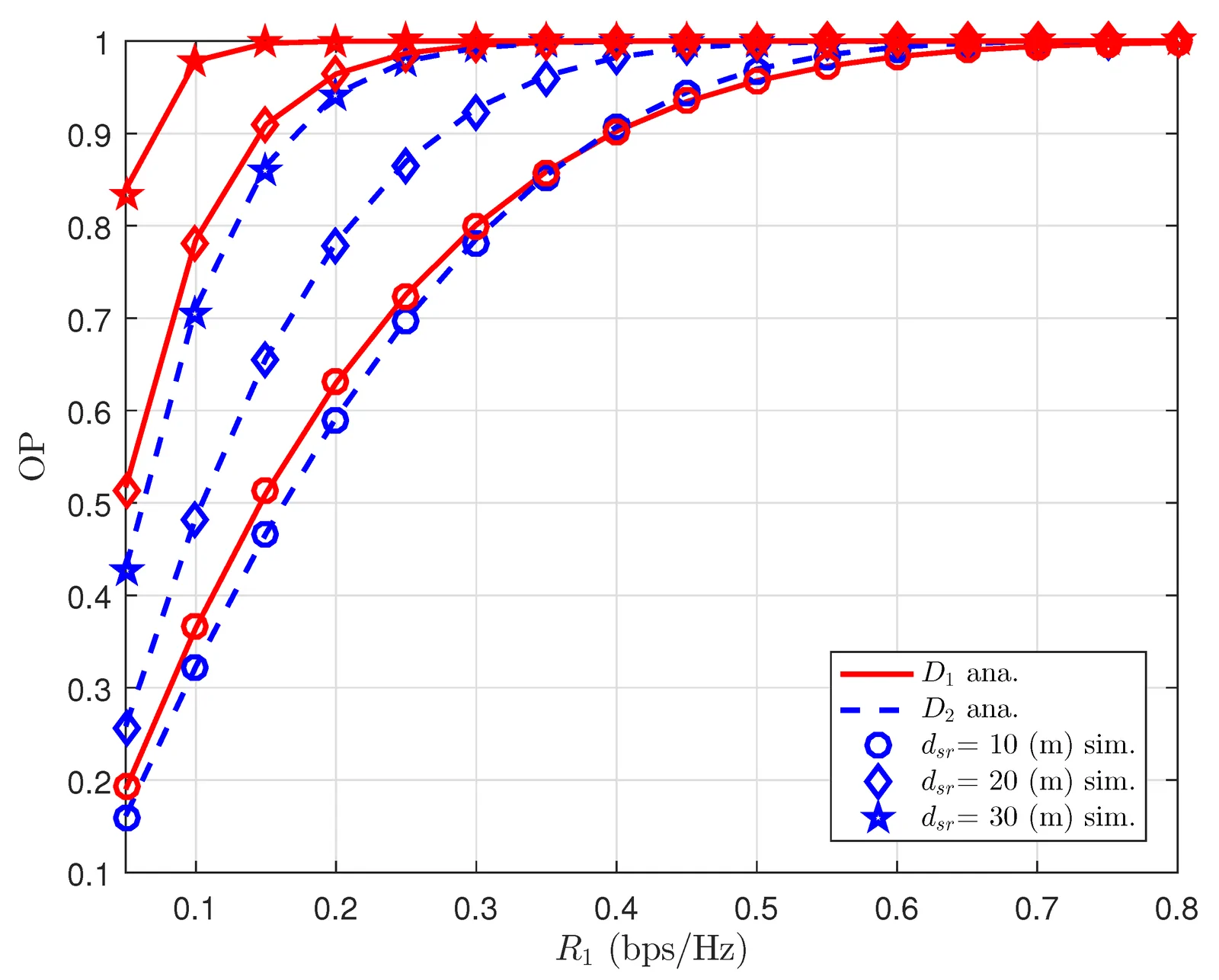
OP versus $R_{1}=R_{2}$ for different $d_{sr}$ .

Figure 5 shows the simulation of OP versus $R_{1}=R_{2}$ for different $d_{sr}$ , i.e., distance between BS−R . Here, we can observe that as the distance increases, the outage performance of the IoT users decreases. Additionally, for higher level target rates assigned in each scenario, the outage performance of each user enters saturation mode. This shows that an optimal distance between the BS−R has a pivotal role in enhancing the performance of the system.

**Figure 6:**
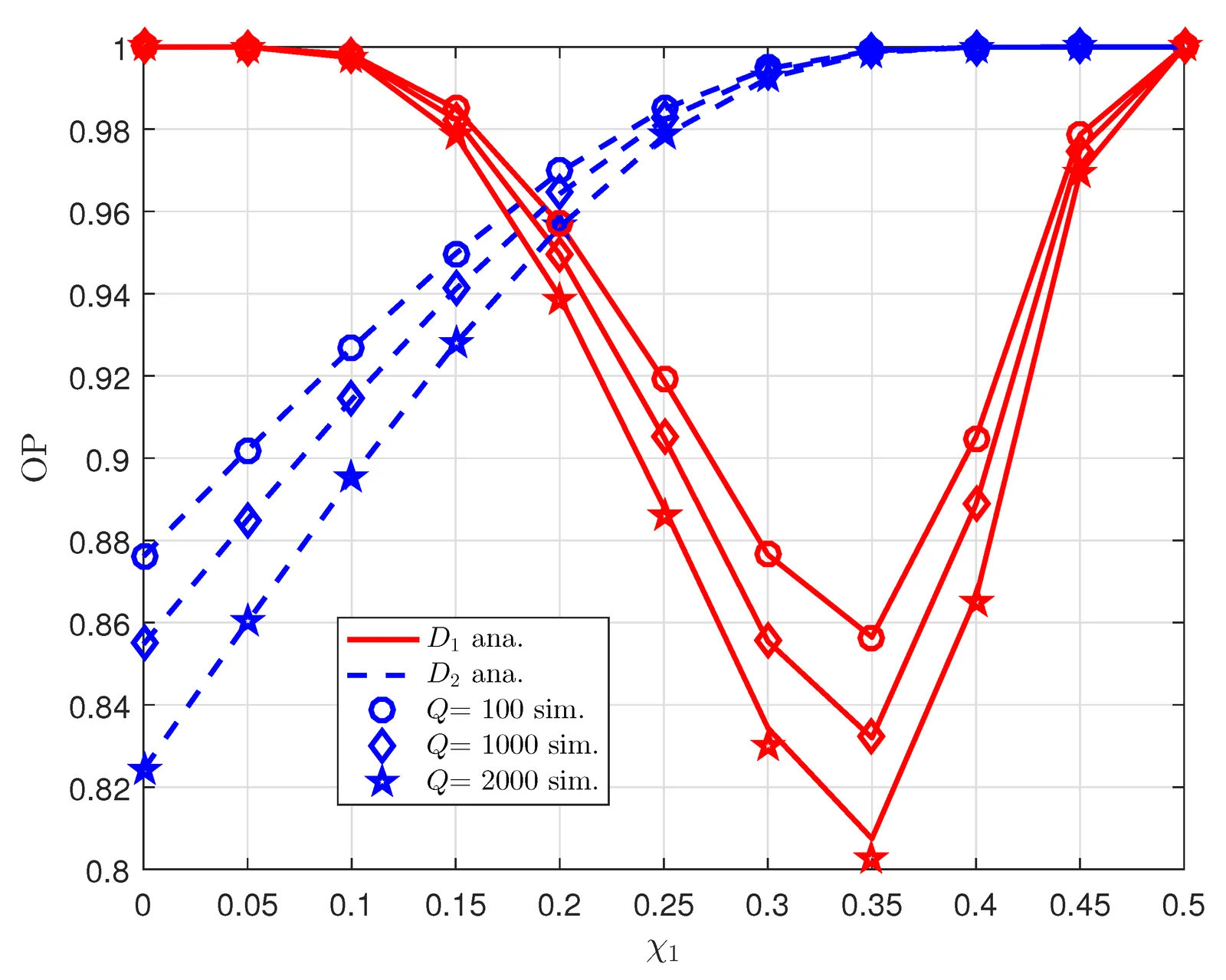
OP versus $\chi_{1}$ for different *Q*.

Figure 6 illustrates the simulation of OP versus $\chi_{1}$ for different *Q*. As we can observe from the simulation, as the power allocation increases for user $D_{1}$ , the performance of the user increases rapidly. However, the number of elements also plays a major role in enhancing the performance of both users. In all cases, the highest number of elements produces the best performance. As we can see, the power coefficients $\chi_{1}$ = $\chi_{2}$ clearly affect the performance significantly. When $\chi_{1}$ increases, the OP of user $D_{2}$ worsens, since it follows the principle of NOMA, while user $D_{1}$ meets the optimal OP values at $\chi_{1}=0.35$ corresponding to the three values of *Q*. This finding provides a numerical way to achieve optimization for OP. It can be seen clearly that Q=2000 exhibits its superiority over the other cases.

**Figure 7:**
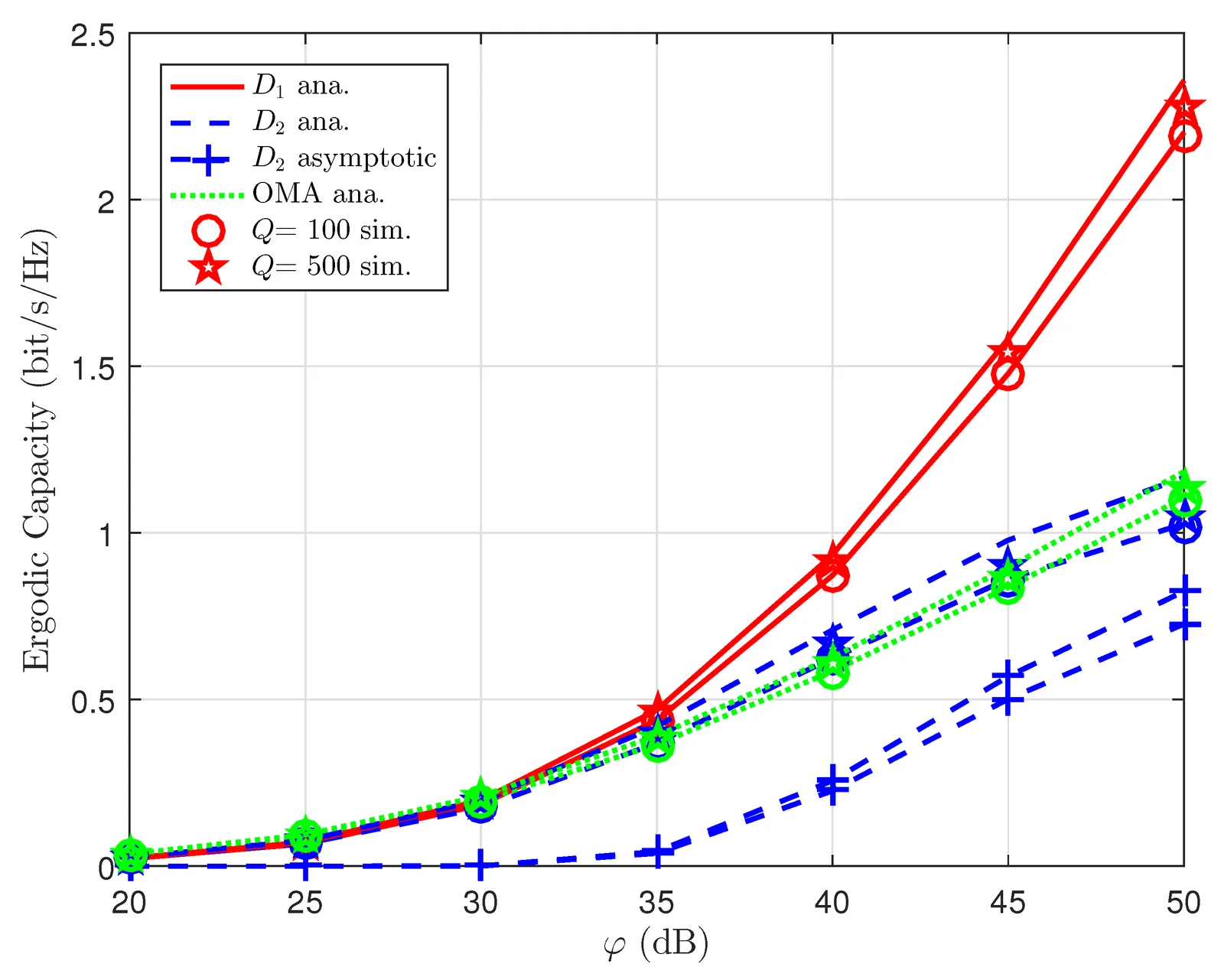
EC versus φ for different *Q* with *G*= *K*= 1000.

**Figure 8:**
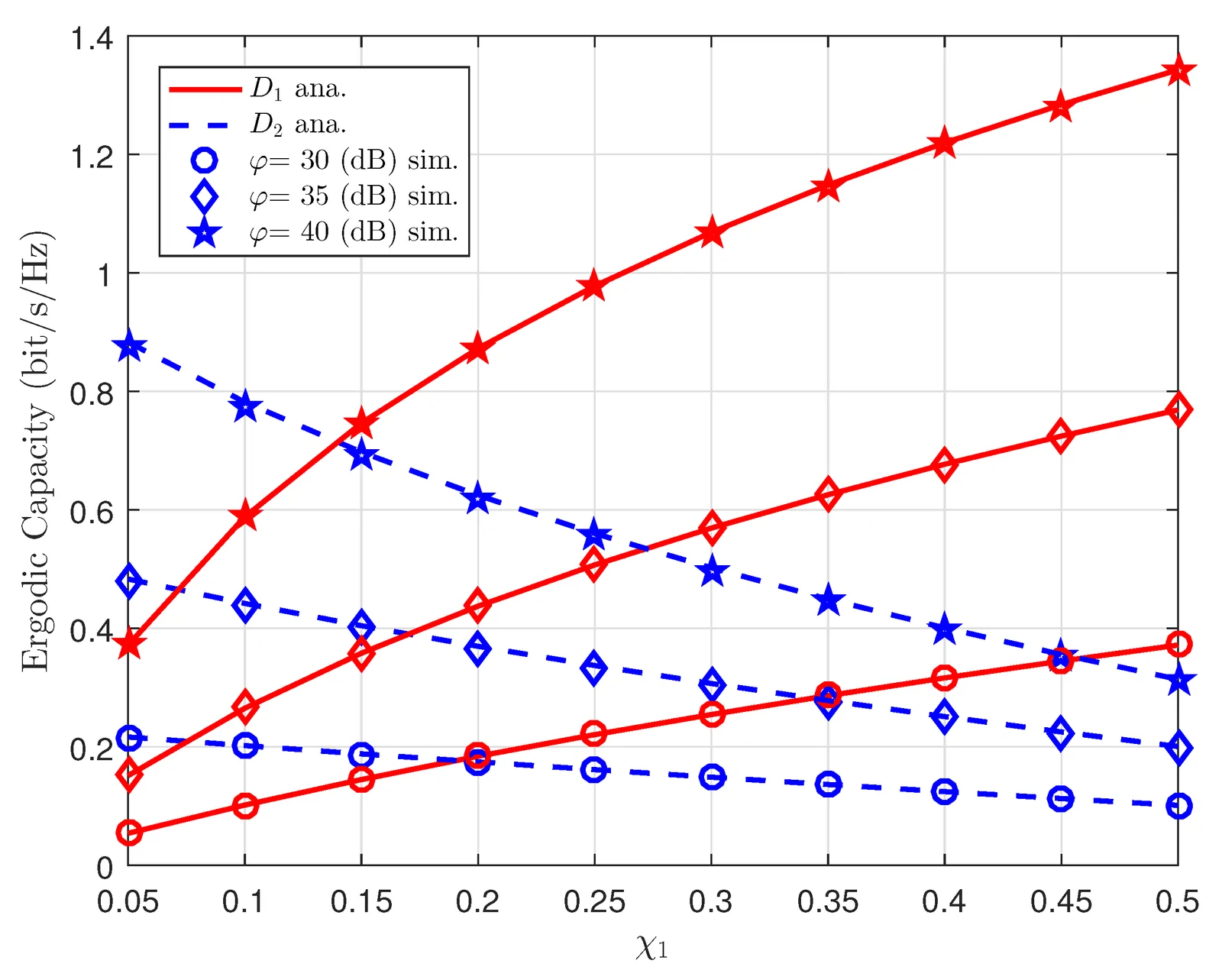
EC versus $\chi_{1}$ for different φ .

Figure 7 shows the simulation of EC versus φ for different *Q* while Figure 8 confirms the impact of the power allocation factor on EC performance. In Figure 7, the simulation demonstrates the performance comparison of NOMA and OMA networks in the proposed model for two different meta-surface elements. Asymptotic analysis of user $D_{2}$ has the worst case EC meanwhile, the near IoT user $D_{1}$ with a higher number of meta-surfaces has the best case EC. Similarly, we can see in Figure 8 that the EC for user $D_{1}$ increases if more power is assigned regardless any values of the average SNR at the source φ .

**Design Guidelines** Below, we provide the following design suggestions extracted from the insightful numerical findings. These analytical points can provide helpful advice in materializing the theoretical insights.

- Considering the curves of OP versus φ for different $\chi_{1}$ , in order to obtain OP of 0.65, φ for the OMA case is required to be 40 (dB). Furthermore, to satisfy the setting of NOMA in the case of $\chi_{1}$ = 0.3 user $D_{1}$ needs φ at 33 (dB) while user $D_{2}$ needs φ = 37 (dB).
- Considering the curves of OP versus φ for different $R_{1}=R_{2}$ , if we want OP to equal 0.5 at $R_{1}=R_{2}$ = 0.5 (bps/Hz), φ for user $D_{1}$ must be 36.5 (dB), and user $D_{2}$ requires φ = 37 (dB). However, increasing target rates to $R_{1}=R_{2}$ = 0.7 (bps/Hz) and maintaining OP at 0.5, these requirements need user $D_{1}$ served by the average SNR at the source φ = 38.5 (dB), and $D_{2}$ corresponds to φ = 40 (dB).
- We need to know how the distances among nodes enact changes in OP with respect to $R_{1}=R_{2}$ , i.e., $d_{sr}$ need to be evaluated. When $d_{sr}$ = 10 (m), the expected OP of 0.5 occurs at user $D_{1}$ for the case $R_{1}=R_{2}$ = 0.15 (bps/Hz), while user $D_{2}$ corresponds to $R_{1}=R_{2}$ = 0.16 (bps/Hz). If we increase $d_{sr}$ to 20 (m), the expected OP of 0.5 for user $D_{1}$ occurs in the case of $R_{1}=R_{2}$ = 0.05 (bps/Hz), and user $D_{2}$ corresponds to $R_{1}=R_{2}$ = 0.1 (bps/Hz).
- We shift our attention to EC versus φ for different *Q* with *G* = *K* = 1000. When *Q* = 100 the EC of 1 (bit/s/Hz) for user $D_{1}$ occurs when φ = 40 (dB), while user $D_{2}$ corresponds to φ = 49 (dB). If the setting of RIS is changed to *Q* = 500, the EC of 1 (bit/s/Hz) required at user $D_{1}$ when φ equals 41 (dB), and φ equals 46 (dB) $D_{2}$ for user $D_{2}$ .

## 8. Conclusions and Future Work

In this paper, we evaluated the performance of an IoT system consisting of RIS and relay when the channels are adopted Rayleigh fading distributions. The closed-form expressions are derived for the RIS-NOMA system and the system’s performance was analyzed in terms of OP and EC. The hybrid scheme demonstrated increased efficiency by comparing practical scenarios via simulations. Simulation analysis was performed using Monte-Carlo methodology, to verify the validity of the obtained expressions. The simulations reveal that the, transmit SNR, number of elements, power allocation, and target rates play a major role in enhancing the performance of IoT users. Meanwhile, the increase in distance and path loss decreases the performance of the system. However, the increase in amplitude coefficient does not show much effect on the performance of users. In future work, we extend our analysis to multiple users to further prove the benefits of NOMA and RIS approaches. Interestingly, we may deploy some machine learning tools to predict the OP and EC performance of users at the base station, which can adjust power allocation factors to better improve performance.

## Figures and Tables

**Table 1:** Comparison between our work and existing works.

Context	[30]	[31]	[32]	[33]	[34]	[35]	[36]	[37]	[38]	[39]	[40]	[41]	Our Work
RIS-NOMA system	×	×	×									×	×
RIS-OMA system	×	×					×	×	×	×			×
Hardware Impairment	×												
Satellite Terrestrial			×	×	×	×							
Hybrid RIS and relay approach										×			×
Rayleigh fading	×					×				×	×	×	×
Perfect SIC and CSI	×					×							×
Optimization			×	×	×		×	×	×	×		×	×
OP Analysis	×	×									×		×
EC Analysis													×
Asymptotic expression	×	×									×	×	×

**Table 2:** Main notations.

Symbol	Description
$\mathrm{Pr}\left(.\right)$	Probability
$F_{X}\left(.\right)$	The cumulative distribution function (CDF) of an RV *X*
$f_{X}\left(.\right)$	The probability density function (PDF) of an RV *X*
$E\left\{.\right\}$	Expectation operator
$\mathrm{Ei}\left\{.\right\}$	The exponential integral function
$\arg\left(x\right)$	The phase of a complex number *x*
$P_{s}$	The transmit power at BS
$P_{r}$	The transmit power at *R*
$z_{i}$	The information symbol of $D_{i}$ with $E\left[{\left|z_{i}\right|}^{2}\right]=1$ , $\left(i=1,2\right)$
$z_{o}$	The information symbol of $D_{o}$ with $E\left[{\left|z_{o}\right|}^{2}\right]=1$
$\chi_{i}$	The corresponding power allocation coefficients of $D_{i}$
$\delta_{r}$	The additive white Gaussian noise (AWGN) at *R* with zero mean and variance of $\sigma_{0}$
$\delta_{i}$	The AWGN at $D_{i}$ with zero mean and variance of $\sigma_{0}$
$\delta_{d}$	The AWGN at $D_{o}$ with zero mean and variance of $\sigma_{0}$
ε	The path loss exponent
$R_{i}$	The target rate at the user $D_{i}$ to detect $z_{i}$
$R_{o}$	The target rate at the user $D_{o}$ to detect $z_{o}$
$g_{sr}$	The complex channel coefficient for the link BS→ *R*
$g_{s,q}$	The complex channel coefficient for the link BS→RI
$g_{r,q}$	The complex channel coefficient for the link RI→R
$h_{r1}$	The complex channel coefficient for the link R→ $D_{1}$
$h_{r,q}$	The complex channel coefficient for the link R→RI
$h_{1,q}$	The complex channel coefficient for the link $RI\to D_{1}$
$h_{r2}$	The complex channel coefficient for the link $R\to D_{2}$
$h_{2,q}$	The complex channel coefficient for the link $RI\to D_{2}$
$h_{rd}$	The complex channel coefficient for the link $R\to D_{o}$
$h_{d,q}$	The complex channel coefficient for the link $RI\to D_{o}$

**Table 3:** Values used in simulations.

Parameters	Notation	Values
NOMA power splitting factors	$\left\{\chi_{1},\chi_{2}\right\}$	$\left\{0.2,0.8\right\}$
The required rates	$R_{1}=R_{2}$ ; $R_{o}$	0.5 (bps/Hz); 0.7 (bps/Hz)
Amplitude reflection coefficient of RIS [45]	Ω = *∂* = ϖ	0.5
Path loss exponent	ε	2.5
The number of meta-surface in RIS	*Q*	100
Distances (Normalized) [41]	$d_{sr}=d_{r1}=d_{i1}=d_{rd}=d_{id}$ ; $d_{si}=d_{ir}=d_{ri}=d_{r2}=d_{i2}$	10 (m); 20 (m)
Channel gains [43]	$\lambda_{sr}=\lambda_{r1}=\lambda_{r2}=\lambda_{rd}$	1
The average SNR at transmitter [41]	φ	30 (dB)

## Data Availability

Not applicable.

## Appendix A

**Proof** **of** **Proposition 1.** From (9), $A_{1}$ can written by

(A1)
$$\begin{array}{cc} A_{1} & =\mathrm{Pr}\left[\left(d_{sr}^{-\varepsilon }{\left|g_{sr}\right|}^{2}+\theta_{1}\Phi_{1}^{2}\right)\chi_{1}\varphi \geq \vartheta_{1}\right]\\ \; & =\mathrm{Pr}\left[{\left|g_{sr}\right|}^{2}\geq \frac{\vartheta_{1}-\chi_{1}\varphi \theta_{1}\Phi_{1}^{2}}{d_{sr}^{-\varepsilon }\chi_{1}\varphi }\right], \end{array}$$

where (A1) must satisfy the condition $\vartheta_{1}-\chi_{1}\varphi \theta_{1}\Phi_{1}^{2}>0\to \Phi_{1}^{2}<\frac{\vartheta_{1}}{\chi_{1}\varphi \theta_{1}}$ . Thus, the CDF and the PDF of all channels can be expressed as $F_{{\left|X\right|}^{2}}\left(x\right)=1-e^{-\frac{x}{\lambda_{X}}}$ and $f_{{\left|X\right|}^{2}}\left(x\right)=\frac{1}{\lambda_{X}}e^{-\frac{x}{\lambda_{X}}}$ [43,44,45]. $A_{1}$ can given as

(A2)
$$\begin{array}{c} A_{1}=\mathrm{Pr}\left[{\left|g_{sr}\right|}^{2}\geq \frac{\vartheta_{1}-\chi_{1}\varphi \theta_{1}\Phi_{1}^{2}}{d_{sr}^{-\varepsilon }\chi_{1}\varphi },\Phi_{1}^{2}<\frac{\vartheta_{1}}{\chi_{1}\varphi \theta_{1}}\right]\\ =\int_{0}^{\frac{\vartheta_{1}}{\chi_{1}\varphi \theta_{1}}}\left[1-F_{{\left|g_{sr}\right|}^{2}}\left(\frac{\vartheta_{1}-\chi_{1}\varphi \theta_{1}x}{d_{sr}^{-\varepsilon }\chi_{1}\varphi }\right)\right]f_{\Phi_{1}^{2}}\left(x\right)dx\\ =\frac{1}{Q}\exp\left(-\frac{\vartheta_{1}}{d_{sr}^{-\varepsilon }\chi_{1}\varphi \lambda_{sr}}\right)\int_{0}^{\frac{\vartheta_{1}}{\chi_{1}\varphi \theta_{1}}}\exp\left[\left(\frac{\theta_{1}}{d_{sr}^{-\varepsilon }\lambda_{sr}}-\frac{1}{Q}\right)x\right]dx\\ =\frac{d_{sr}^{-\varepsilon }\lambda_{sr}}{Q\theta_{1}-d_{sr}^{-\varepsilon }\lambda_{sr}}\exp\left(-\frac{\vartheta_{1}}{d_{sr}^{-\varepsilon }\chi_{1}\varphi \lambda_{sr}}\right)\left[\exp\left[\left(\frac{\theta_{1}}{d_{sr}^{-\varepsilon }\lambda_{sr}}-\frac{1}{Q}\right)\frac{\vartheta_{1}}{\chi_{1}\varphi \theta_{1}}\right]-1\right]. \end{array}$$

From (9), $A_{2}$ can written by

(A3)
$$\begin{array}{c} A_{2}=\mathrm{Pr}\left(\begin{array}{c} \frac{\left(d_{r1}^{-\varepsilon }{\left|h_{r1}\right|}^{2}+\theta_{2}\Phi_{2}^{2}\right)\chi_{2}\varphi }{\left(d_{r1}^{-\varepsilon }{\left|h_{r1}\right|}^{2}+\theta_{2}\Phi_{2}^{2}\right)\chi_{1}\varphi +1}\geq \vartheta_{2},\left(d_{r1}^{-\varepsilon }{\left|h_{r1}\right|}^{2}+\theta_{2}\Phi_{2}^{2}\right)\chi_{1}\varphi \geq \vartheta_{1} \end{array}\right)\\ =\mathrm{Pr}\left(\begin{array}{c} d_{r1}^{-\varepsilon }{\left|h_{r1}\right|}^{2}+\theta_{2}\Phi_{2}^{2}\geq \frac{\vartheta_{2}}{\left(\chi_{2}-\chi_{1}\vartheta_{2}\right)\varphi },d_{r1}^{-\varepsilon }{\left|h_{r1}\right|}^{2}+\theta_{2}\Phi_{2}^{2}\geq \frac{\vartheta_{1}}{\chi_{1}\varphi } \end{array}\right)\\ =\mathrm{Pr}\left(d_{r1}^{-\varepsilon }{\left|h_{r1}\right|}^{2}+\theta_{2}\Phi_{2}^{2}\geq \max\left(\frac{\vartheta_{2}}{\left(\chi_{2}-\chi_{1}\vartheta_{2}\right)\varphi },\frac{\vartheta_{1}}{\chi_{1}\varphi }\right)\right). \end{array}$$

We let $\nu =\max\left(\frac{\vartheta_{2}}{\left(\chi_{2}-\chi_{1}\vartheta_{2}\right)\varphi },\frac{\vartheta_{1}}{\chi_{1}\varphi }\right)$ and (A3) must satisfy the condition $\nu -\theta_{2}\Phi_{2}^{2}>0\to \Phi_{2}^{2}<\frac{\nu }{\theta_{2}}$ . $A_{2}$ can be calculated as follows

(A4)
$$\begin{array}{c} A_{2}=\mathrm{Pr}\left({\left|h_{r_{1}}\right|}^{2}\geq \frac{\nu -\theta_{2}\Phi_{2}^{2}}{d_{r1}^{-\varepsilon }},\Phi_{2}^{2}<\frac{\nu }{\theta_{2}}\right)\\ =\int_{0}^{\frac{\nu }{\theta_{2}}}\left(1-F_{{\left|h_{r_{1}}\right|}^{2}}\left(\frac{\nu -\theta_{2}x}{d_{r1}^{-\varepsilon }}\right)\right)f_{\Phi_{2}^{2}}\left(x\right)dx\\ =\frac{1}{Q}\exp\left(-\frac{\nu }{d_{r1}^{-\varepsilon }\lambda_{r1}}\right)\int_{0}^{\frac{\nu }{\theta_{2}}}\exp\left(\left(\frac{\theta_{2}}{d_{r1}^{-\varepsilon }\lambda_{r1}}-\frac{1}{Q}\right)x\right)dx\\ =\frac{d_{r1}^{-\varepsilon }\lambda_{r1}}{\theta_{2}Q-d_{r1}^{-\varepsilon }\lambda_{r1}}\exp\left(-\frac{\nu }{d_{r1}^{-\varepsilon }\lambda_{r1}}\right)\left[\exp\left(\left(\frac{\theta_{2}}{d_{r1}^{-\varepsilon }\lambda_{r1}}-\frac{1}{Q}\right)\frac{\nu }{\theta_{2}}\right)-1\right]. \end{array}$$

Combining (A2), (A4) into (9), we can obtain (10). This completes the proof. □

## Appendix B

**Proof** **of** **Proposition 2.** From (11), $B_{1}$ can written by

(A5)
$$\begin{array}{c} B_{1}=\mathrm{Pr}\left(\frac{\left(d_{sr}^{-\varepsilon }{\left|g_{sr}\right|}^{2}+\theta_{1}\Phi_{1}^{2}\right)\chi_{2}\varphi }{\left(d_{sr}^{-\varepsilon }{\left|g_{sr}\right|}^{2}+\theta_{1}\Phi_{1}^{2}\right)\chi_{1}\varphi +1}\geq \vartheta_{2}\right)\\ =\mathrm{Pr}\left(d_{sr}^{-\varepsilon }{\left|g_{sr}\right|}^{2}+\theta_{1}\Phi_{1}^{2}\geq \frac{\vartheta_{2}}{\left(\chi_{2}-\chi_{1}\vartheta_{2}\right)\varphi }\right)\\ =\mathrm{Pr}\left({\left|g_{sr}\right|}^{2}\geq \frac{\vartheta_{2}-\left(\chi_{2}-\chi_{1}\vartheta_{2}\right)\varphi \theta_{1}\Phi_{1}^{2}}{\left(\chi_{2}-\chi_{1}\vartheta_{2}\right)\varphi d_{sr}^{-\varepsilon }}\right), \end{array}$$

where (A5) must satisfy the condition $\chi_{2}>\chi_{1}\vartheta_{2}$ and $\Phi_{1}^{2}<\frac{\vartheta_{2}}{\left(\chi_{2}-\chi_{1}\vartheta_{2}\right)\varphi \theta_{1}}$ . $B_{1}$ can be obtained as

(A6)
$$\begin{array}{c} B_{1}=\mathrm{Pr}\left(\begin{array}{c} {\left|g_{sr}\right|}^{2}\geq \frac{\vartheta_{2}-\left(\chi_{2}-\chi_{1}\vartheta_{2}\right)\varphi \theta_{1}\Phi_{1}^{2}}{\left(\chi_{2}-\chi_{1}\vartheta_{2}\right)\varphi d_{sr}^{-\varepsilon }},\Phi_{1}^{2}<\frac{\vartheta_{2}}{\left(\chi_{2}-\chi_{1}\vartheta_{2}\right)\varphi \theta_{1}} \end{array}\right)\\ =\int_{0}^{\frac{\vartheta_{2}}{\left(\chi_{2}-\chi_{1}\vartheta_{2}\right)\varphi \theta_{1}}}\left(1-F_{{\left|g_{sr}\right|}^{2}}\left(\frac{\vartheta_{2}-\left(\chi_{2}-\chi_{1}\vartheta_{2}\right)\varphi \theta_{1}x}{\left(\chi_{2}-\chi_{1}\vartheta_{2}\right)\varphi d_{sr}^{-\varepsilon }}\right)\right)f_{\Phi_{1}^{2}}\left(x\right)dx\\ =\frac{1}{Q}\exp\left(-\frac{\vartheta_{2}}{\left(\chi_{2}-\chi_{1}\vartheta_{2}\right)\varphi d_{sr}^{-\varepsilon }\lambda_{sr}}\right)\int_{0}^{\frac{\vartheta_{2}}{\left(\chi_{2}-\chi_{1}\vartheta_{2}\right)\varphi \theta_{1}}}\exp\left(\left(\frac{\theta_{1}}{d_{sr}^{-\varepsilon }\lambda_{sr}}-\frac{1}{Q}\right)x\right)dx\\ =\frac{d_{sr}^{-\varepsilon }\lambda_{sr}}{\theta_{1}Q-d_{sr}^{-\varepsilon }\lambda_{sr}}\exp\left(-\frac{\vartheta_{2}}{\left(\chi_{2}-\chi_{1}\vartheta_{2}\right)\varphi d_{sr}^{-\varepsilon }\lambda_{sr}}\right)\left[\exp\left(\left(\frac{\theta_{1}}{d_{sr}^{-\varepsilon }\lambda_{sr}}-\frac{1}{Q}\right)\frac{\vartheta_{2}}{\left(\chi_{2}-\chi_{1}\vartheta_{2}\right)\varphi \theta_{1}}\right)-1\right]. \end{array}$$

From (11), $B_{2}$ can be rewritten as

(A7)
$$\begin{array}{c} B_{2}=\mathrm{Pr}\left(\frac{\left(d_{r2}^{-\varepsilon }{\left|h_{r2}\right|}^{2}+\theta_{3}\Phi_{3}^{2}\right)\chi_{2}\varphi }{\left(d_{r2}^{-\varepsilon }{\left|h_{r2}\right|}^{2}+\theta_{3}\Phi_{3}^{2}\right)\chi_{1}\varphi +1}\geq \vartheta_{2}\right)\\ =\mathrm{Pr}\left(d_{r2}^{-\varepsilon }{\left|h_{r2}\right|}^{2}+\theta_{3}\Phi_{3}^{2}\geq \frac{\vartheta_{2}}{\left(\chi_{2}-\chi_{1}\vartheta_{2}\right)\varphi }\right)\\ =\mathrm{Pr}\left({\left|h_{r2}\right|}^{2}\geq \frac{\vartheta_{2}-\left(\chi_{2}-\chi_{1}\vartheta_{2}\right)\varphi \theta_{3}\Phi_{3}^{2}}{\left(\chi_{2}-\chi_{1}\vartheta_{2}\right)\varphi d_{r2}^{-\varepsilon }}\right), \end{array}$$

where (A7) must satisfy the condition $\chi_{2}>\chi_{1}\vartheta_{2}$ and $\Phi_{3}^{2}<\frac{\vartheta_{2}}{\left(\chi_{2}-\chi_{1}\vartheta_{2}\right)\varphi \theta_{3}}$ . $B_{2}$ is given by

(A8)
$$\begin{array}{c} B_{2}=\mathrm{Pr}\left(\begin{array}{c} {\left|h_{r2}\right|}^{2}\geq \frac{\vartheta_{2}-\left(\chi_{2}-\chi_{1}\vartheta_{2}\right)\varphi \theta_{3}\Phi_{3}^{2}}{\left(\chi_{2}-\chi_{1}\vartheta_{2}\right)\varphi d_{r2}^{-\varepsilon }},\Phi_{3}^{2}<\frac{\vartheta_{2}}{\left(\chi_{2}-\chi_{1}\vartheta_{2}\right)\varphi \theta_{3}} \end{array}\right)\\ =\int_{0}^{\frac{\vartheta_{2}}{\left(\chi_{2}-\chi_{1}\vartheta_{2}\right)\varphi \theta_{3}}}\left(1-F_{{\left|h_{r2}\right|}^{2}}\left(\frac{\vartheta_{2}-\left(\chi_{2}-\chi_{1}\vartheta_{2}\right)\varphi \theta_{3}x}{\left(\chi_{2}-\chi_{1}\vartheta_{2}\right)\varphi d_{r2}^{-\varepsilon }}\right)\right)f_{\Phi_{3}^{2}}\left(x\right)dx\\ =\frac{1}{Q}\exp\left(-\frac{\vartheta_{2}}{\left(\chi_{2}-\chi_{1}\vartheta_{2}\right)\varphi d_{r2}^{-\varepsilon }\lambda_{r2}}\right)\int_{0}^{\frac{\vartheta_{2}}{\left(\chi_{2}-\chi_{1}\vartheta_{2}\right)\varphi \theta_{3}}}\exp\left(\left(\frac{\theta_{3}}{d_{r2}^{-\varepsilon }\lambda_{r2}}-\frac{1}{Q}\right)x\right)dx\\ =\frac{d_{r2}^{-\varepsilon }\lambda_{r2}}{\theta_{3}Q-d_{r2}^{-\varepsilon }\lambda_{r2}}\exp\left(-\frac{\vartheta_{2}}{\left(\chi_{2}-\chi_{1}\vartheta_{2}\right)\varphi d_{r2}^{-\varepsilon }\lambda_{r2}}\right)\left[\exp\left(\left(\frac{\theta_{3}}{d_{r2}^{-\varepsilon }\lambda_{r2}}-\frac{1}{Q}\right)\frac{\vartheta_{2}}{\left(\chi_{2}-\chi_{1}\vartheta_{2}\right)\varphi \theta_{3}}\right)-1\right]. \end{array}$$

Combining (A6), (A8) into (11), we can obtain (12). This completes the proof. □

## Appendix C

**Proof** **of** **Proposition 3.** From (13), $F_{\Xi_{1}}\left(t\right)$ can be written as

(A9)
$$\begin{array}{cc} F_{\Xi_{1}}\left(t\right) & =\mathrm{Pr}\left(\min\left(\gamma_{r}^{z_{1}},\gamma_{D_{1}}^{z_{1}}\right)<t\right)\\ \; & =1-\left(1-F_{\gamma_{r}^{z_{1}}}\left(t\right)\right)\left(1-F_{\gamma_{D_{1}}^{z_{1}}}\left(t\right)\right). \end{array}$$

From (A9), $F_{\gamma_{r}^{z_{1}}}\left(t\right)$ can be calculated as

(A10)
$$\begin{array}{c} F_{\gamma_{r}^{z_{1}}}\left(t\right)=1-\mathrm{Pr}\left[\left(d_{sr}^{-\varepsilon }{\left|g_{sr}\right|}^{2}+\theta_{1}\Phi_{1}^{2}\right)\chi_{1}\varphi \geq t\right]\\ =1-\mathrm{Pr}\left[{\left|g_{sr}\right|}^{2}\geq \frac{t-\chi_{1}\varphi \theta_{1}\Phi_{1}^{2}}{d_{sr}^{-\varepsilon }\chi_{1}\varphi }\right]\\ =1-\int_{0}^{\infty }\left[1-F_{{\left|g_{sr}\right|}^{2}}\left(\frac{t-\chi_{1}\varphi \theta_{1}x}{d_{sr}^{-\varepsilon }\chi_{1}\varphi }\right)\right]f_{\Phi_{1}^{2}}\left(x\right)dx\\ =1-\frac{1}{Q}\exp\left(-\frac{t}{d_{sr}^{-\varepsilon }\chi_{1}\varphi \lambda_{sr}}\right)\int_{0}^{\infty }\exp\left[-\left(\frac{1}{Q}-\frac{\theta_{1}}{d_{sr}^{-\varepsilon }\lambda_{sr}}\right)x\right]dx\\ =1-\frac{d_{sr}^{-\varepsilon }\lambda_{sr}}{d_{sr}^{-\varepsilon }\lambda_{sr}-Q\theta_{1}}\exp\left(-\frac{t}{d_{sr}^{-\varepsilon }\chi_{1}\varphi \lambda_{sr}}\right). \end{array}$$

Then, $F_{\gamma_{D_{1}}^{z_{1}}}\left(t\right)$ can be written as

(A11)
$$\begin{array}{c} F_{\gamma_{D_{1}}^{z_{1}}}\left(t\right)=1-\mathrm{Pr}\left(\left(d_{r1}^{-\varepsilon }{\left|h_{r1}\right|}^{2}+\theta_{2}\Phi_{2}^{2}\right)\chi_{1}\varphi \geq t\right)\\ =1-\mathrm{Pr}\left[{\left|h_{r1}\right|}^{2}\geq \frac{t-\chi_{1}\varphi \theta_{2}\Phi_{2}^{2}}{d_{r1}^{-\varepsilon }\chi_{1}\varphi }\right]\\ =1-\int_{0}^{\infty }\left[1-F_{{\left|h_{r1}\right|}^{2}}\left(\frac{t-\chi_{1}\varphi \theta_{2}x}{d_{r1}^{-\varepsilon }\chi_{1}\varphi }\right)\right]f_{\Phi_{2}^{2}}\left(x\right)dx\\ =1-\frac{1}{Q}\exp\left(-\frac{t}{d_{r1}^{-\varepsilon }\chi_{1}\varphi \lambda_{r1}}\right)\int_{0}^{\infty }\exp\left[-\left(\frac{1}{Q}-\frac{\theta_{2}}{d_{r1}^{-\varepsilon }\lambda_{r1}}\right)x\right]dx\\ =1-\frac{d_{r1}^{-\varepsilon }\lambda_{r1}}{d_{r1}^{-\varepsilon }\lambda_{r1}-Q\theta_{2}}\exp\left(-\frac{t}{d_{r1}^{-\varepsilon }\chi_{1}\varphi \lambda_{r1}}\right). \end{array}$$

From (A10) and (A11) into (A9), $F_{\Xi_{1}}\left(t\right)$ can be written as

(A12)
$$F_{\Xi_{1}}\left(t\right)=1-\mu_{1}\mu_{2}\exp\left(-\xi t\right),$$

where $\mu_{1}=\frac{d_{sr}^{-\varepsilon }\lambda_{sr}}{d_{sr}^{-\varepsilon }\lambda_{sr}-Q\theta_{1}}$ , $\mu_{2}=\frac{d_{r1}^{-\varepsilon }\lambda_{r1}}{d_{r1}^{-\varepsilon }\lambda_{r1}-Q\theta_{2}}$ , $\xi =\frac{1}{d_{sr}^{-\varepsilon }\chi_{1}\varphi \lambda_{sr}}+\frac{1}{d_{r1}^{-\varepsilon }\chi_{1}\varphi \lambda_{r1}}$ . From (A12) into (13). Based on [50] (Equation (3.352.4)) and applying some polynomial expansion manipulations, ${\bar{ℜ}}_{1}$ can be written as

(A13)
$$\begin{array}{c} {\bar{ℜ}}_{1}=\frac{1}{2\ln2}\int_{0}^{\infty }\frac{\mu_{1}\mu_{2}}{1+t}\exp\left(-\xi t\right)dt\\ =-\frac{1}{2\ln2}\mu_{1}\mu_{2}\exp\left(\xi \right)\mathrm{Ei}\left(-\xi \right). \end{array}$$

The proof is completed. □

## Appendix D

**Proof** **of** **Proposition 4.** From (15), $F_{\Xi_{2}}\left(t\right)$ can be written as

(A14)
$$\begin{array}{cc} F_{\Xi_{2}}\left(t\right) & =\mathrm{Pr}\left(\min\left(\gamma_{r}^{z_{2}},\gamma_{D_{1}}^{z_{2}},\gamma_{D_{2}}^{z_{2}}\right)<t\right)\\ \; & =1-\left(1-F_{\gamma_{r}^{z_{2}}}\left(t\right)\right)\left(1-F_{\gamma_{D_{1}}^{z_{2}}}\left(t\right)\right)\left(1-F_{\gamma_{D_{2}}^{z_{2}}}\left(t\right)\right). \end{array}$$

From (A14), $F_{\gamma_{r}^{z_{2}}}\left(t\right)$ can be expressed as

(A15)
$$\begin{array}{c} F_{\gamma_{r}^{z_{2}}}\left(t\right)=1-\mathrm{Pr}\left(\frac{\left(d_{sr}^{-\varepsilon }{\left|g_{sr}\right|}^{2}+\theta_{1}\Phi_{1}^{2}\right)\chi_{2}\varphi }{\left(d_{sr}^{-\varepsilon }{\left|g_{sr}\right|}^{2}+\theta_{1}\Phi_{1}^{2}\right)\chi_{1}\varphi +1}\geq t\right)\\ =1-\mathrm{Pr}\left({\left|g_{sr}\right|}^{2}\geq \frac{t-\left(\chi_{2}-\chi_{1}t\right)\varphi \theta_{1}\Phi_{1}^{2}}{\left(\chi_{2}-\chi_{1}t\right)\varphi d_{sr}^{-\varepsilon }}\right)\\ =1-\int_{0}^{\infty }\left(1-F_{{\left|g_{sr}\right|}^{2}}\left(\frac{t-\left(\chi_{2}-\chi_{1}t\right)\varphi \theta_{1}x}{\left(\chi_{2}-\chi_{1}t\right)\varphi d_{sr}^{-\varepsilon }}\right)\right)f_{\Phi_{1}^{2}}\left(x\right)dx\\ =1-\frac{1}{Q}\exp\left(-\frac{t}{\left(\chi_{2}-\chi_{1}t\right)\varphi d_{sr}^{-\varepsilon }\lambda_{sr}}\right)\int_{0}^{\infty }\exp\left(-\left(\frac{1}{Q}-\frac{\theta_{1}}{d_{sr}^{-\varepsilon }\lambda_{sr}}\right)x\right)dx\\ =1-\mu_{1}\exp\left(-\frac{t}{\left(\chi_{2}-\chi_{1}t\right)\varphi d_{sr}^{-\varepsilon }\lambda_{sr}}\right). \end{array}$$

Then, $F_{\gamma_{D_{1}}^{z_{2}}}\left(t\right)$ can be expressed as

(A16)
$$\begin{array}{c} F_{\gamma_{D_{1}}^{z_{2}}}\left(t\right)=1-\mathrm{Pr}\left(\frac{\left(d_{r1}^{-\varepsilon }{\left|h_{r1}\right|}^{2}+\theta_{2}\Phi_{2}^{2}\right)\chi_{2}\varphi }{\left(d_{r1}^{-\varepsilon }{\left|h_{r1}\right|}^{2}+\theta_{2}\Phi_{2}^{2}\right)\chi_{1}\varphi +1}\geq t\right)\\ =1-\mathrm{Pr}\left({\left|h_{r1}\right|}^{2}\geq \frac{t-\left(\chi_{2}-\chi_{1}t\right)\varphi \theta_{2}\Phi_{2}^{2}}{\left(\chi_{2}-\chi_{1}t\right)\varphi d_{r1}^{-\varepsilon }}\right)\\ =1-\int_{0}^{\infty }\left(1-F_{{\left|h_{r1}\right|}^{2}}\left(\frac{t-\left(\chi_{2}-\chi_{1}t\right)\varphi \theta_{2}\Phi_{2}^{2}}{\left(\chi_{2}-\chi_{1}t\right)\varphi d_{r1}^{-\varepsilon }}\right)\right)f_{\Phi_{2}^{2}}\left(x\right)dx\\ =1-\frac{1}{Q}\exp\left(-\frac{t}{\left(\chi_{2}-\chi_{1}t\right)\varphi d_{r1}^{-\varepsilon }\lambda_{r1}}\right)\int_{0}^{\infty }\exp\left(-\left(\frac{1}{Q}-\frac{\theta_{2}}{d_{r1}^{-\varepsilon }\lambda_{r1}}\right)x\right)dx\\ =1-\mu_{2}\exp\left(-\frac{t}{\left(\chi_{2}-\chi_{1}t\right)\varphi d_{r1}^{-\varepsilon }\lambda_{r1}}\right). \end{array}$$

Next, $F_{\gamma_{D_{2}}^{z_{2}}}\left(t\right)$ can be written as

(A17)
$$\begin{array}{c} F_{\gamma_{D_{2}}^{z_{2}}}\left(t\right)=1-\mathrm{Pr}\left(\frac{\left(d_{r2}^{-\varepsilon }{\left|h_{r2}\right|}^{2}+\theta_{3}\Phi_{3}^{2}\right)\chi_{2}\varphi }{\left(d_{r2}^{-\varepsilon }{\left|h_{r2}\right|}^{2}+\theta_{3}\Phi_{3}^{2}\right)\chi_{1}\varphi +1}\geq t\right)\\ =1-\mathrm{Pr}\left({\left|h_{r2}\right|}^{2}\geq \frac{t-\left(\chi_{2}-\chi_{1}t\right)\varphi \theta_{3}\Phi_{3}^{2}}{\left(\chi_{2}-\chi_{1}t\right)\varphi d_{r2}^{-\varepsilon }}\right)\\ =1-\int_{0}^{\infty }\left(1-F_{{\left|h_{r2}\right|}^{2}}\left(\frac{t-\left(\chi_{2}-\chi_{1}t\right)\varphi \theta_{3}x}{\left(\chi_{2}-\chi_{1}t\right)\varphi d_{r2}^{-\varepsilon }}\right)\right)f_{\Phi_{3}^{2}}\left(x\right)dx\\ =1-\frac{1}{Q}\exp\left(-\frac{t}{\left(\chi_{2}-\chi_{1}t\right)\varphi d_{r2}^{-\varepsilon }\lambda_{r2}}\right)\int_{0}^{\infty }\exp\left(-\left(\frac{1}{Q}-\frac{\theta_{3}}{d_{r2}^{-\varepsilon }\lambda_{r2}}\right)x\right)dx\\ =1-\frac{d_{r2}^{-\varepsilon }\lambda_{r2}}{d_{r2}^{-\varepsilon }\lambda_{r2}-\theta_{3}Q}\exp\left(-\frac{t}{\left(\chi_{2}-\chi_{1}t\right)\varphi d_{r2}^{-\varepsilon }\lambda_{r2}}\right). \end{array}$$

From (A15)–(A17) into (A14), $F_{\Xi_{2}}\left(t\right)$ can be expressed as

(A18)
$$F_{\Xi_{2}}\left(t\right)=1-\mu_{1}\mu_{2}\mu_{3}\exp\left(-\frac{\phi t}{\left(\chi_{2}-\chi_{1}t\right)}\right),$$

where $\mu_{3}=\frac{d_{r2}^{-\varepsilon }\lambda_{r2}}{d_{r2}^{-\varepsilon }\lambda_{r2}-\theta_{3}Q}$ , $\phi =\frac{1}{\varphi d_{sr}^{-\varepsilon }\lambda_{sr}}+\frac{1}{\varphi d_{r1}^{-\varepsilon }\lambda_{r1}}+\frac{1}{\varphi d_{r2}^{-\varepsilon }\lambda_{r2}}$ . From (A18) into (15), ${\bar{ℜ}}_{2}$ is given by

(A19)
$${\bar{ℜ}}_{2}=\frac{1}{2\ln2}\int_{0}^{\frac{\chi_{2}}{\chi_{1}}}\frac{\mu_{1}\mu_{2}\mu_{3}}{1+t}\exp\left(-\frac{\phi t}{\left(\chi_{2}-\chi_{1}t\right)}\right)dt.$$

We let $w=\frac{\chi_{1}}{\chi_{2}}t$ , ${\bar{ℜ}}_{2}$ is written as

(A20)
$${\bar{ℜ}}_{2}=\frac{1}{2\ln2}\int_{0}^{1}\frac{\chi_{1}\chi_{2}\mu_{1}\mu_{2}\mu_{3}}{\left(\chi_{1}+\chi_{2}w\right)\chi_{1}}\exp\left(-\frac{\phi \chi_{2}w}{\left(\chi_{2}-\chi_{2}w\right)\chi_{1}}\right)dw.$$

The proof is completed. □

## Appendix E

**Proof** **of** **Proposition 5.** From (22), $A_{1,o}$ can be written as

(A21)
$$\begin{array}{c} A_{1,o}=\mathrm{Pr}\left[\left(d_{sr}^{-\varepsilon }{\left|g_{sr}\right|}^{2}+\theta_{1}\Phi_{1}^{2}\right)\varphi \geq \vartheta_{o}\right]\\ =\mathrm{Pr}\left[{\left|g_{sr}\right|}^{2}\geq \frac{\vartheta_{o}-\varphi \theta_{1}\Phi_{1}^{2}}{d_{sr}^{-\varepsilon }\varphi },\Phi_{1}^{2}<\frac{\vartheta_{o}}{\varphi \theta_{1}}\right]\\ =\int_{0}^{\frac{\vartheta_{o}}{\varphi \theta_{1}}}\left[1-F_{{\left|g_{sr}\right|}^{2}}\left(\frac{\vartheta_{o}-\varphi \theta_{1}x}{d_{sr}^{-\varepsilon }\varphi }\right)\right]f_{\Phi_{1}^{2}}\left(x\right)dx\\ =\frac{d_{sr}^{-\varepsilon }\lambda_{sr}}{Q\theta_{1}-d_{sr}^{-\varepsilon }\lambda_{sr}}\exp\left(-\frac{\vartheta_{o}}{d_{sr}^{-\varepsilon }\varphi \lambda_{sr}}\right)\left[\exp\left[\left(\frac{\theta_{1}}{d_{sr}^{-\varepsilon }\lambda_{sr}}-\frac{1}{Q}\right)\frac{\vartheta_{o}}{\varphi \theta_{1}}\right]-1\right]. \end{array}$$

Then, $A_{2,o}$ can written by

(A22)
$$\begin{array}{c} A_{2,o}=\mathrm{Pr}\left(\left(d_{rd}^{-\varepsilon }{\left|h_{rd}\right|}^{2}+\theta_{i,o}\Phi_{i,o}^{2}\right)\varphi \geq \vartheta_{o}\right)\\ =\mathrm{Pr}\left[{\left|h_{rd}\right|}^{2}\geq \frac{\vartheta_{o}-\varphi \theta_{i,o}\Phi_{i,o}^{2}}{d_{rd}^{-\varepsilon }\varphi },\Phi_{i,o}^{2}<\frac{\vartheta_{o}}{\varphi \theta_{i,o}}\right]\\ =\int_{0}^{\frac{\vartheta_{o}}{\varphi \theta_{i,o}}}\left[1-F_{{\left|h_{rd}\right|}^{2}}\left(\frac{\vartheta_{o}-\varphi \theta_{i,o}x}{d_{rd}^{-\varepsilon }\varphi }\right)\right]f_{\Phi_{i,o}^{2}}\left(x\right)dx\\ =\frac{d_{rd}^{-\varepsilon }\lambda_{rd}}{Q\theta_{i,o}-d_{rd}^{-\varepsilon }\lambda_{rd}}\exp\left(-\frac{\vartheta_{o}}{d_{rd}^{-\varepsilon }\varphi \lambda_{rd}}\right)\left[\exp\left[\left(\frac{\theta_{i,o}}{d_{rd}^{-\varepsilon }\lambda_{rd}}-\frac{1}{Q}\right)\frac{\vartheta_{o}}{\varphi \theta_{i,o}}\right]-1\right]. \end{array}$$

Combining (A21), (A22) into (22), we can obtain (23). It completes the proof. □

## Appendix F

**Proof** **of** **Proposition 6.** From (24), $F_{\Xi_{O}}\left(t\right)$ can written by

(A23)
$$\begin{array}{cc} F_{\Xi_{O}}\left(t\right) & =\mathrm{Pr}\left(\min\left(\gamma_{r,o},\gamma_{D_{o}}\right)<t\right)\\ \; & =1-\left(1-F_{\gamma_{r,o}}\left(t\right)\right)\left(1-F_{\gamma_{D_{o}}}\left(t\right)\right). \end{array}$$

From (A23), $F_{\gamma_{r,o}}\left(t\right)$ can given as

(A24)
$$\begin{array}{c} F_{\gamma_{r,o}}\left(t\right)=1-\mathrm{Pr}\left[\left(d_{sr}^{-\varepsilon }{\left|g_{sr}\right|}^{2}+\theta_{1}\Phi_{1}^{2}\right)\varphi \geq t\right]\\ =1-\mathrm{Pr}\left[{\left|g_{sr}\right|}^{2}\geq \frac{t-\varphi \theta_{1}\Phi_{1}^{2}}{d_{sr}^{-\varepsilon }\varphi }\right]\\ =1-\int_{0}^{\infty }\left[1-F_{{\left|g_{sr}\right|}^{2}}\left(\frac{t-\varphi \theta_{1}x}{d_{sr}^{-\varepsilon }\varphi }\right)\right]f_{\Phi_{1}^{2}}\left(x\right)dx\\ =1-\mu_{1}\exp\left(-\frac{t}{d_{sr}^{-\varepsilon }\varphi \lambda_{sr}}\right). \end{array}$$

Then, $F_{\gamma_{D_{o}}}\left(t\right)$ can given as

(A25)
$$\begin{array}{c} F_{\gamma_{D_{o}}}\left(t\right)=\mathrm{Pr}\left(\left(d_{rd}^{-\varepsilon }{\left|h_{rd}\right|}^{2}+\theta_{i,o}\Phi_{i,o}^{2}\right)\varphi \geq t\right)\\ =\mathrm{Pr}\left[{\left|h_{rd}\right|}^{2}\geq \frac{t-\varphi \theta_{i,o}\Phi_{i,o}^{2}}{d_{rd}^{-\varepsilon }\varphi }\right]\\ =\int_{0}^{\infty }\left[1-F_{{\left|h_{rd}\right|}^{2}}\left(\frac{t-\varphi \theta_{i,o}x}{d_{rd}^{-\varepsilon }\varphi }\right)\right]f_{\Phi_{i,o}^{2}}\left(x\right)dx\\ =\frac{d_{rd}^{-\varepsilon }\lambda_{rd}}{d_{rd}^{-\varepsilon }\lambda_{rd}-Q\theta_{i,o}}\exp\left(-\frac{t}{d_{rd}^{-\varepsilon }\varphi \lambda_{rd}}\right). \end{array}$$

From (A24) and (A25) into (A23), $F_{\Xi_{O}}\left(t\right)$ can written by

(A26)
$$F_{\Xi_{O}}\left(t\right)=1-\frac{\mu_{1}d_{rd}^{-\varepsilon }\lambda_{rd}}{d_{rd}^{-\varepsilon }\lambda_{rd}-Q\theta_{i,o}}\exp\left(-\frac{t}{d_{sr}^{-\varepsilon }\varphi \lambda_{sr}}-\frac{t}{d_{rd}^{-\varepsilon }\varphi \lambda_{rd}}\right).$$

From (A26) into (24), ${\bar{ℜ}}_{O}$ can given as

(A27)
$$\begin{array}{c} {\bar{ℜ}}_{O}=\frac{1}{6\ln2}\frac{\mu_{1}d_{rd}^{-\varepsilon }\lambda_{rd}}{d_{rd}^{-\varepsilon }\lambda_{rd}-Q\theta_{i,o}}\int_{0}^{\infty }\frac{1}{1+t}\exp\left(-\left(\frac{1}{d_{sr}^{-\varepsilon }\varphi \lambda_{sr}}+\frac{1}{d_{rd}^{-\varepsilon }\varphi \lambda_{rd}}\right)t\right)dt\\ =-\frac{1}{6\ln2}\frac{\mu_{1}d_{rd}^{-\varepsilon }\lambda_{rd}}{d_{rd}^{-\varepsilon }\lambda_{rd}-Q\theta_{i,o}}\exp\left(\frac{1}{d_{sr}^{-\varepsilon }\varphi \lambda_{sr}}+\frac{1}{d_{rd}^{-\varepsilon }\varphi \lambda_{rd}}\right)\mathrm{Ei}\left(-\left(\frac{1}{d_{sr}^{-\varepsilon }\varphi \lambda_{sr}}+\frac{1}{d_{rd}^{-\varepsilon }\varphi \lambda_{rd}}\right)\right), \end{array}$$

where (A27) can be obtained by using [50] (Equation (3.352.4)). The proof is completed. □
