# Correction: Dang et al. Empowering Intelligent Surfaces and User Pairing for IoT Relaying Systems: Outage Probability and Ergodic Capacity Performance. *Sensors* 2022, *22*, 6576

**DOI:** 10.3390/s26154886

**Published:** 2026-08-03

**Authors:** Huu-Phuc Dang, Minh-Sang Van Nguyen, Dinh-Thuan Do, Minh-Hoa Nguyen, Minh-Triet Pham, Anh-Tuan Kim

**Affiliations:** 1 Electrical-Electronics Department, School of Engineering and Technology, Tra Vinh University, Tra Vinh 87000, Vietnam; hoatvu@tvu.edu.vn (M.-H.N.); minhtriet@tvu.edu.vn (M.-T.P.); katuan@tvu.edu.vn (A.-T.K.); 2 Faculty of Electronics Technology, Industrial University of Ho Chi Minh City (IUH), Ho Chi Minh City 70000, Vietnam; nguyenvanminhsang@iuh.edu.vn; 3 Department of Computer Science and Information Engineering, College of Information and Electrical Engineering, Asia University, Taichung 41354, Taiwan; dodinhthuan@asia.edu.tw

## Error in Table

In the original publication [1], there was a mistake in Table 1. The authors mistakenly cited Reference 39 in Table 1. Therefore, Reference 39 should be deleted from Table 1. The updated Table 1 appears below:

## References Correction

Reference 39 was removed. With this correction, the order of some references has been adjusted accordingly.

The authors state that the scientific conclusions are unaffected. This correction was approved by the Academic Editor. The original publication has also been updated.

## Figures and Tables

**Table 1:** Comparison between our work and existing works.

Context	[30]	[31]	[32]	[33]	[34]	[35]	[36]	[37]	[38]	[39]	[40]	[41]	Our Work
RIS-NOMA system	×	×	×									×	×
RIS-OMA system	×	×					×	×	×	×			×
Hardware Impairment	×												
Satellite Terrestrial			×	×	×	×							
Hybrid RIS and relay approach										×			×
Rayleigh fading	×					×				×	×	×	×
Perfect SIC and CSI	×					×							×
Optimization			×	×	×		×	×	×	×		×	×
OP Analysis	×	×									×		×
EC Analysis													×
Asymptotic expression	×	×									×	×	×
